# *Ex situ* population dynamics of Brazilian parrots based on zoo, trade and conservation data

**DOI:** 10.1098/rsos.261135

**Published:** 2026-11-11

**Authors:** Isabel Reis Baltazar, Ivana Schork, Luiza Figueiredo Passos, Gabriela de Araújo Porto Ramos, Maria Rita Silverio Pires, Cristiano Schetini de Azevedo

**Affiliations:** ^1^ Universidade Federal de Ouro Preto, Ouro Preto, Brazil; ^2^ Department of Animal and Agriculture, Hartpury University and Hartpury College, Hartpury, Gloucester, UK; ^3^ Liverpool John Moores University, Liverpool, UK

**Keywords:** trade, *ex situ* conservation, parrots, reproductive success, zoos

## Abstract

Zoos contribute to *ex situ* conservation of taxa affected by habitat loss and wildlife trade, including parrots, but their success in maintaining viable populations remains unclear. This study assessed the role of zoos in the *ex situ* maintenance and reproduction of Brazilian parrot species over 2004–2024 using Zoological Information Management System demographic records, Convention on International Trade in Endangered Species of Wild Fauna and Flora (CITES) trade data and International Union for Conservation of Nature Red List categories. Generalized linear mixed models, generalized additive models and generalized additive mixed models tested effects of population growth, threat status, sex ratio and extinction risk in relation to trade intensity. *Ex situ* populations showed no consistent demographic growth. Hatching rates declined over time, and *per capita* net population change was near zero or negative for most species, with populations often sustained by acquisitions rather than reproduction. More threatened species did not show higher reproductive success under captive management. Sex ratio was associated with population dynamics, with more favourable outcomes near balanced ratios. International trade was positively associated with extinction risk, with species at higher threat levels appearing more frequently in trade records. These findings show that captive population growth is uneven across species and highlight the need for integrated demographic, welfare, genetic and trade management to improve conservation outcomes.

## 1. Introduction

Psittacidae, the family encompassing macaws, parrots, parakeets and conures, is among the most threatened avian groups worldwide [1,2]. Its vulnerability is primarily driven by intensified anthropogenic pressures, including habitat destruction, landscape modification and the loss of nesting resources associated with deforestation, agricultural expansion and altered fire regimes linked to agricultural land management [3,4]. These threats are worsened by the illegal wildlife trade, a major driver of population declines across the tropics, especially among charismatic, highly sought-after parrots [5,6]. This pressure is particularly pronounced in Brazil, a global hot spot for psittacine diversity, where endemic species face increasing habitat degradation and ongoing trafficking [7,8].

Brazil is therefore a particularly informative case study for examining the demographic performance of parrots maintained in zoological institutions. It supports the world's highest number of parrot species, including several endemic taxa, as well as a substantial number of species threatened with extinction [2,9,10]. The broad range of extinction risk within the parrot family provides an opportunity to examine whether species with different conservation statuses show distinct patterns of population dynamics under human care. Understanding these patterns can help assess the potential of zoological populations to support the longer-term conservation of threatened parrot species.

In this context, zoological institutions are crucial to the *ex situ* conservation of threatened parrots. They maintain assurance populations, develop captive breeding programmes and support long-term genetic and demographic management. In addition to caring for wildlife under human care, zoos play a vital role in researching species’ biology, behaviour, welfare and reproduction, thereby improving captive management and informing broader conservation efforts [11,12].

Recent advances in global biodiversity databases have expanded the capacity to evaluate the conservation role of zoos. The Zoological Information Management System (ZIMS), hosted by Species360, provides one of the most comprehensive global repositories of demographic and husbandry records for animals under human care, enabling assessments of birth rates, mortality, sex ratios and population trends across institutions [13,14]. When combined with complementary international datasets such as the Convention on International Trade in Endangered Species of Wild Fauna and Flora (CITES) [15], which documents cross-border movements of listed species, and the International Union for Conservation of Nature (IUCN) Red List, which provides the global standard for extinction risk assessment, these data allow *ex situ* population dynamics to be linked to global trade patterns and conservation status [16–19].

Despite the strategic importance often attributed to captive breeding programmes, quantitative evidence on whether zoo parrot populations are demographically self-sustaining over time remains limited. Although some studies have quantified life-history and reproductive parameters in captive parrots and examined their relationship with conservation status, these analyses have generally been separate from large-scale assessments of international trade [20–22]. Consequently, it remains unclear whether these populations are sustained mainly by internal reproduction or by continued acquisitions, and how these processes differ among species with varying conservation statuses. Although demographic performance is an important component of population sustainability, long-term viability also depends on genetic management, particularly in small or isolated captive populations [23,24].

To address this gap, this study evaluates the demographic and reproductive performance of Brazilian parrot species maintained in zoological institutions by integrating demographic data from ZIMS with international trade records from the CITES database. Specifically, we (i) analyse temporal trends in hatchings from 2004 to 2024, (ii) evaluate population growth and reproductive patterns in captive populations, (iii) examine the relationship between sex ratios and demographic stability, and (iv) assess how international trade patterns vary with extinction risk.

We tested four hypotheses on the dynamics of captive populations and international trade. First, we predicted that zoo-managed populations would show positive demographic performance, with hatchings exceeding deaths and resulting in growth, driven by structured management and breeding programmes. Second, we expected threatened species to perform better in captivity, with those classified as Vulnerable, Endangered or Critically Endangered showing higher reproductive output or population growth than less threatened species, potentially reflecting greater management and conservation attention. Third, we predicted that the sex ratio would influence population stability, with ratios closer to parity associated with greater stability or growth. Finally, we expected international trade to decline with higher extinction risk, driven by regulatory restrictions and conservation constraints.

## 2. Material and methods

### 2.1. Database sources and species selection

Data for the study were compiled from three primary sources: ZIMS, CITES and the IUCN Red List, spanning the 20-year period from 2004 to 2024. The analysis included all Brazilian parrot species recorded in ZIMS (63 species across 22 genera; supplementary material, S1).

ZIMS provided records of individuals held in zoological institutions, including sex, hatchings, deaths, acquisitions and dispositions. Data were obtained via a structured search on the Species360 platform using a ZIMS for Education subscription held by Liverpool John Moores University.

Information on international trade was obtained from the CITES database, including annual import and export volumes (restricted to live individuals; feathers and eggs excluded), the principal trading countries for each transaction and species listed under appendices I and II. Species threat status across the study period was derived from the IUCN Red List. Data from CITES and the IUCN Red List were obtained from publicly available reports.

Demographic, trade and conservation status data were integrated into a single dataset to examine relationships among captive population dynamics, extinction risk and international trade.

As all data were pre-existing and accessed through institutional or public sources, no ethical approval was required.

## 3. Data analysis

### 3.1. *Ex situ* population dynamics

Reproductive dynamics and population trends of Brazilian parrot species maintained under human care were assessed using annual hatching records organized by species and year. Exploratory analyses visualized temporal patterns. Data were organized by species and year, and exploratory analyses were conducted to visualize temporal patterns. Species were used as the comparative units because the study aimed to characterize demographic patterns across the 63 Brazilian parrot species represented in the dataset. This approach recognizes that species differ substantially in life history, reproductive biology, generation length and husbandry requirements, which may contribute to heterogeneity in demographic patterns among taxa. Species identity was included as a random effect to account for repeated observations and between-species heterogeneity, while temporal dependence was considered where appropriate. Line graphs illustrated annual hatchings per species, alongside genus-level averages to highlight broader taxonomic trends.

Differences in hatching rates across IUCN threat categories were tested using a generalized linear mixed model (GLMM) with a negative binomial distribution with linear parameterization (NB1), which was selected to account for overdispersion in the count data. To identify the best-fitting distribution and correlation structure, we compared eight candidate models: Poisson, NB1, NB2, zero-inflated NB1 and NB2, and NB1/NB2 models with a first-order autoregressive [AR(1)] correlation structure within species. Model selection was based on Akaike information criteria (AIC), Baysian information criteria and DHARMa residual diagnostics (see §3.5. ‘Model diagnostics’). The final model used a negative binomial distribution (NB1) with an AR(1) correlation structure within species, which provided the best fit (AIC = 5058.5; ΔAIC > 80 compared with models without AR(1)). The response variable was the annual number of hatchings, with IUCN threat category and standardized year (Year_c) included as fixed effects to assess differences among threat categories while accounting for temporal trends across the study period. Year was standardized (mean = 0, s.d. = 1) to improve model convergence and interpretability [25–27].

For repeated observations over time, the AR(1) correlation structure was included to account for temporal dependence within species. The AR(1) structure assumes that correlations decline with temporal distance, so observations closer in time are expected to be more similar [28,29]. This approach has also been used to account for correlation among repeated longitudinal measurements [30,31]. Year was treated as a factor (Year_f) to define the temporal ordering required by the AR(1) structure. A log-transformed total population size was included as an offset to account for differences among species and estimate hatching rates relative to the number of individuals available for reproduction [32,33]. Model assumptions were assessed using simulation-based residual diagnostics (see §3.5. ‘Model diagnostics’ and the supplementary material, S2).

### 3.2. Demographic metrics

Demographic viability of captive populations was assessed using species-level annual records obtained from ZIMS. The following metrics were calculated: population growth, hatching rate, mortality rate, acquisition rate and sex ratio. Calculations assumed that demographic events (hatchings, deaths, acquisitions and dispositions) occurred during year *t*, influencing the population at the end of the previous year (*t* − 1) (table 1).

**Table 1.** Equations used to estimate demographic metrics of Brazilian parrot species maintained in captivity. (Demographic events (occurred throughout year *t*) were assumed to affect the population at the end of the previous year (*t* − 1)).

metric	equation	description
population growth	Δ*N* = *N_t_* − *N_t − 1_*	absolute change in the number of individuals between consecutive years
hatching rate	hatching*_t_*/*N_t − 1_*	number of hatchings in year *t* relative to the total population in the previous year
mortality rate	deaths*_t_*/*N_t − 1_*	number of deaths in year *t* relative to the total population in the previous year
acquisition rate	acquisitions*_t_*/*N_t − 1_*	number of newly acquired individuals in year *t* relative to the total population in the previous year
sex ratio	*N*♂/(*N*♂+*N*♀)	proportion of males among individuals with known sex

Net population change was calculated as follows:

$$\begin{array}{r} NetChange=hatchings+acquisitions-deaths-dispositions. \end{array}$$

This metric represents the annual balance between individuals entering and leaving the captive population. Positive NetChange values indicate a net increase in the number of individuals, whereas negative values indicate a net decrease, with the balance reflecting both reproductive output and movements into and out of the population.

To enable comparisons among species with differing population sizes, this metric was standardized as follows:

$$\begin{array}{r} NetChange\_pc=NetChange/total. \end{array}$$

NetChange_pc represents the proportional annual change relative to the population size at the beginning of the period. Thus, positive values indicate population growth relative to the number of individuals present, values close to zero indicate approximate demographic stability, and negative values indicate population decline. This standardization allows comparison of demographic change among species with different captive population sizes.

### 3.3. Population dynamics models

Three complementary modelling approaches were used to assess temporal patterns in population dynamics and the influence of demographic variables on population change: (i) global generalized additive models (GAMs), (ii) genus-level GAMs, and (iii) species-specific GAMs and generalized additive mixed models (GAMMs).

First, global GAMs were fitted to the entire dataset to explore overall temporal patterns in *per capita* population change (NetChange_pc). The global models included a global smooth for year, a factor-smooth interaction between year and species and a random-effect smooth for species. Four specifications were tested: Gaussian and scaled-t (family = scat) distributions, with and without IUCN threat category as a fixed effect. However, none of the global specifications met DHARMa diagnostic assumptions (uniformity *p* < 0.001 in all cases; 14–26 outliers detected), reflecting the intrinsic heterogeneity of NetChange_pc across species. Global GAMs were therefore reported for descriptive purposes only, and inference was based on genus-level and species-level models.

Second, genus-level GAMs were fitted to characterize *per capita* population change for each of the 22 genera, using year as a smooth term. Species identity was included as a random-effect smooth when a genus contained more than one species and omitted when the genus was represented by a single species. To accommodate the heterogeneity of *per capita* population change across taxa, two specifications were tested for genus-level models: (i) a continuous specification with a scaled-t distribution (family = scat), robust to outliers and heavy tails; and (ii) a binary specification with a binomial distribution, in which the response was whether NetChange_pc > 0. For each genus, the specification with the best combination of adjusted *R*^2^ and residual uniformity was selected for inference.

Third, species-specific GAMs were fitted for all 59 species that met the minimum data requirements (≥ 5 observations and ≥ 2 years), using a single smooth term for year (s(Year, *k* = 8)).

Finally, GAMMs were used to assess the influence of specific demographic variables on population change. Response variables included *per capita* population change (NetChange_pc). Explanatory variables included sex ratio and IUCN threat category (H3), and year was modelled as a smooth term to capture potential nonlinear temporal trends. Taxonomic identity was included as a random effect to account for non-independence of repeated observations and heterogeneity among taxa.

Model assumptions were assessed as described in §3.5. ‘Model diagnostics’ (supplementary material, S2).

### 3.4. Trade dynamics and extinction risk

The relationship between extinction risk and international trade was analysed by integrating CITES trade records and ZIMS population data. Annual trade volume for each species was calculated as the sum of trading events:

$$\begin{array}{r} total\_trade=importer\_reported\_quantity+exporter\_reported\_quantity. \end{array}$$

Captive population size was log-transformed using log1p to accommodate zero values.

International trade dynamics were modelled using Gaussian GAMMs, with log-transformed trade volume as the response variable. Explanatory variables included threat level (treated as an ordinal gradient based on IUCN categories; Least Concern (LC) = 1, Near Threatened (NT) = 2, Vunerable (VU) = 3, Endangered (EN) = 4, Critically Endangered (CR) = 5, Extinct in the Wild (EW) = 6), log-transformed captive population size, trading regions and CITES purpose and source codes (treated as categorical factors) describing the declared purpose of the transaction and biological origin of the specimens. Year was included as a smooth term to capture nonlinear temporal variation in trade across the study period, with the basis dimension selected to balance flexibility and overfitting. Taxonomic identity (taxon) was included as a random effect to account for non-independence of repeated observations and heterogeneity among taxa. Model assumptions were assessed as described in §3.5. ‘Model diagnostics’ (supplementary material, S2).

### 3.5. Model diagnostics

Comprehensive diagnostic procedures were applied to all GLMMs, GAMs and GAMMs, and full outputs are provided in the supplementary material, S2, tables S1–S11 and figures S1–S6. Five approaches were used.

First, simulation-based residual diagnostics were performed using the DHARMa package [34] which generates simulated residuals from the fitted model and compares observed residual patterns to those expected under the assumed distribution. For each model, we tested uniformity (a significant *p*-value indicates deviation from the expected residual distribution), dispersion, outliers (via bootstrap, *n* = 1000) and quantile deviations. Models were considered to have adequate residual structure when uniformity *p* > 0.05.

Second, basis dimension adequacy for smooth terms was assessed using gam.check() from mgcv [35]. Values of *K*-index > 0.90 were considered adequate. For random-effect smooths (bs = ‘re’), the *K*-index test is not defined and returns NA, which is expected behaviour of the mgcv package.

Third, concurvity was assessed using concurvity() from mgcv with full = TRUE. High concurvity between s(Year) and s(Year, Species) is expected because both terms share the year covariate; it does not invalidate inference but may inflate uncertainty.

Fourth, temporal autocorrelation in residuals was assessed by computing the mean autocorrelation function at lag-1 (ACF-1) across species or genera, since DHARMa's testTemporalAutocorrelation() requires balanced panels and our data were unbalanced.

Fifth, for the trade GAMM (Gaussian family), residual normality was assessed using Anderson–Darling, Shapiro–Wilk (on random subsamples owing to the *n* ≤ 5000 limit) and Kolmogorov–Smirnov tests, complemented by skewness, kurtosis and visual inspection of quantile–quantile plots and histograms. Because formal normality tests are highly sensitive at large sample sizes, we interpreted departures from normality using effect sizes and visual inspection.

Overall, model assumptions were adequately met for the GLMM of hatchings (AIC = 5058.5 for the final model with AR(1); marginal *R*^2^ = 0.288; conditional *R*^2^ = 0.378 for the model without AR(1), as the AR(1) structure prevented reliable computation of Nakagawa's *R*^2^; supplementary material, S2, table S1), for genus-level GAMs (all 22 genera had at least one specification with adequate residual uniformity; supplementary material, S2, tables S5–S7) and for the trade GAMM (adjusted *R*^2^ = 0.455; supplementary material, S2, tables S9–S12). The GAMM relating sex ratio to *per capita* population change met assumptions only partially (significant smooth term but low overall fit and residual non-uniformity) and is reported with caution (supplementary material, S2, table S8). The global GAMs of *per capita* population change did not meet assumptions for any specification, reflecting the intrinsic heterogeneity of NetChange_pc across species; these models are reported for descriptive purposes only, and inference is based on genus-level models (supplementary material, S2, tables S2–S4). The supplementary material S2 (supplementary material, table S0) summarizes diagnostic outcomes.

All analyses were performed in R 4.5.1. [36]. GLMMs were fitted using glmmTMB [37], whereas GAMs and GAMMs were fitted using mgcv with restricted maximum likelihood (REML) estimation [35]. The DHARMa package [34] was used for simulation-based residual diagnostics, and the performance package [38] was used to compute marginal and conditional *R*^2^ (Nakagawa's *R*^2^) for mixed models.

Adjusted model estimates were obtained using emmeans [39]. Data manipulation and preparation were conducted using dplyr and tidyr [40,41], and visualizations were produced using ggplot2 [42]. Additional workflows included GAMM component extraction using gratia [43], assignment of continental regions using countrycode [44], data cleaning with janitor [45] and the visualization of adjusted model effects using visreg [46].

All data generated or analysed in this study are available online via the Mendeley Data repository [47].

## 4. Results

### 4.1. Demographic performance of captive populations (H1)

Between 2004 and 2024, the number of Brazilian parrot species in zoological institutions varied among years, while hatching rates declined over time (Year_c: incidence rate ratio (IRR) = 0.80, 95% confidence interval (CI): 0.73–0.87, *p* < 0.001), with no consistent population growth among the most threatened species. Model-based predictions for the mean year (2014), standardized to one unit of total exposure, indicated estimated hatching rates of 0.056 for CR species (95% CI: 0.030–0.108), 0.055 for EN (95% CI: 0.040–0.077), 0.062 for VU species (95% CI: 0.044–0.088), 0.058 for NT species (95% CI: 0.041–0.083) and 0.040 for LC species (95% CI: 0.033–0.050). The estimate for EW species was close to zero (6.9 × 10⁻⁸), with a highly imprecise CI, and was therefore not interpreted further (figures 1–3).

**Figure 1.**
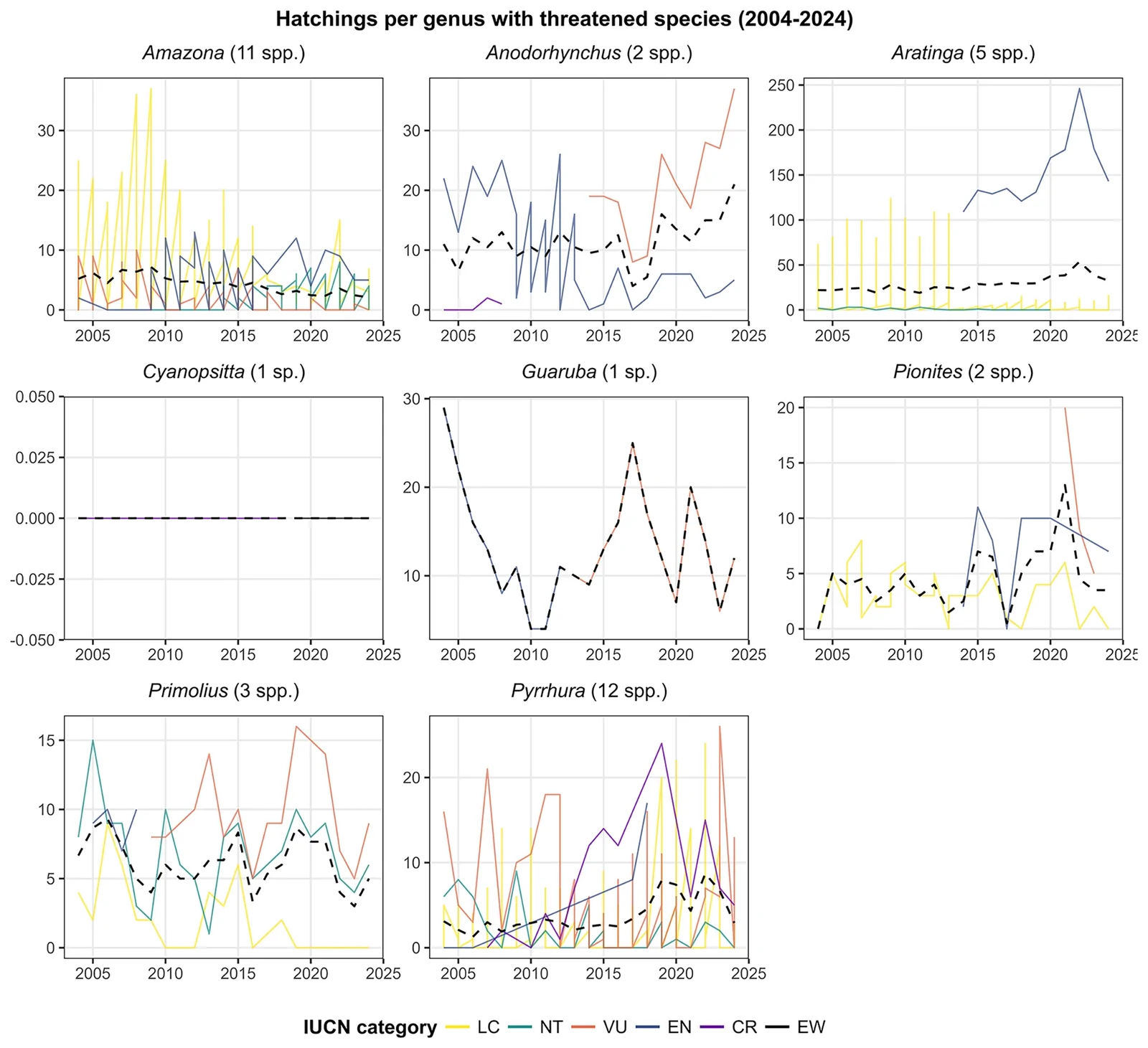
Number of hatchings per threatened genus of Brazilian parrot species maintained under human care, 2004–2024. Lines show individual species; the dashed black line indicates the genus mean. Eight genera containing threatened species (VU, EN, CR and EW) are included. Most threatened genera had low and irreygular reproductive output, suggesting that captive populations rely on management interventions rather than reproduction alone. Colours indicate IUCN categories (LC, yellow; NT, midnight blue; VU, pink; EN, Oxford blue; CR, purple; EW, dark blue).

**Figure 2.**
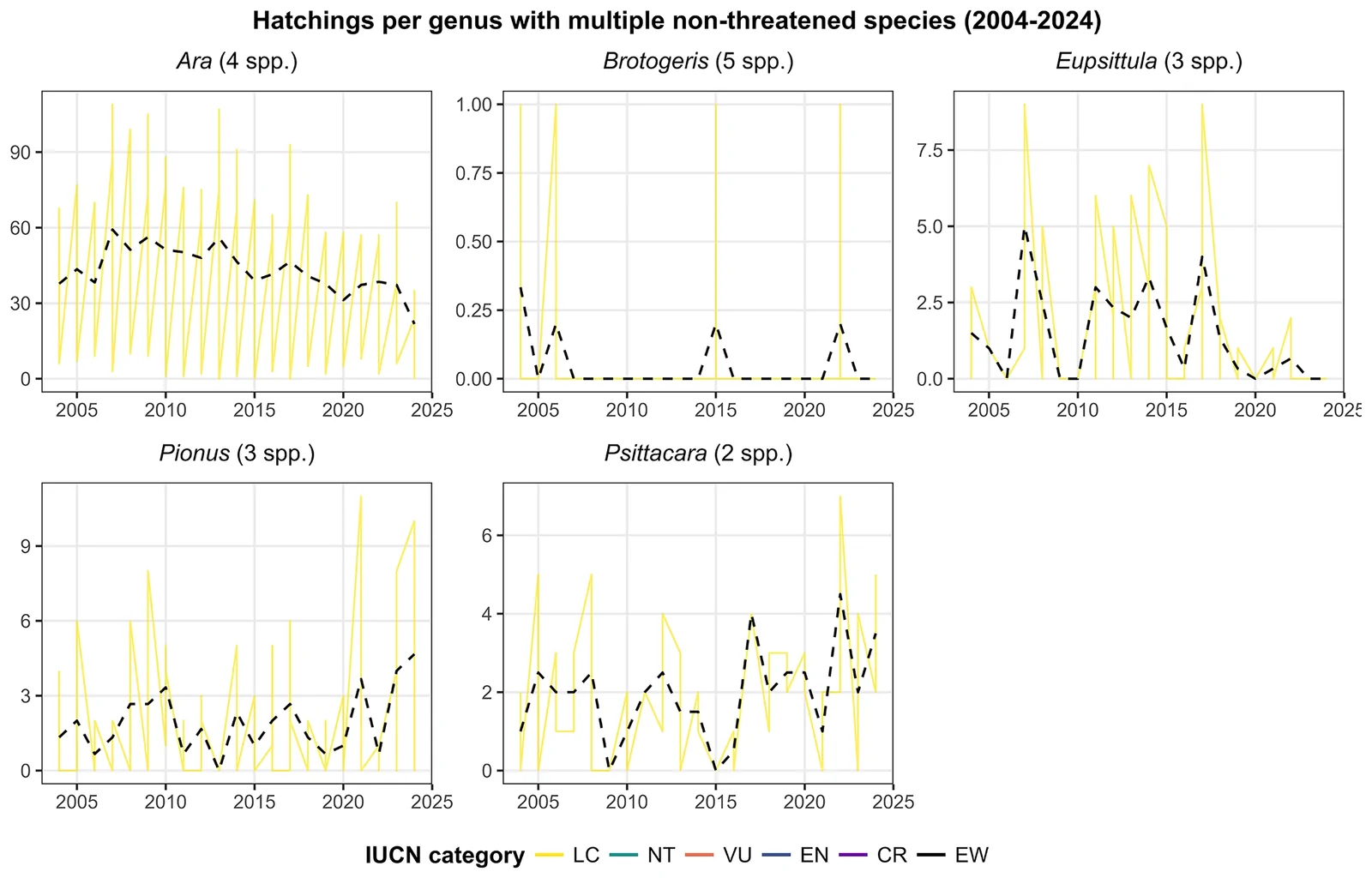
Number of hatchings per multispecies non-threatened genus, 2004–2024. Lines show individual species; dashed black line indicates the genus mean. Five genera with LC species and ≥ 2 species are shown. *Ara* had the highest number of hatchings; *Brotogeris* and *Eupsittula* had low and variable output. Colours indicate IUCN categories (LC, yellow; NT, midnight blue; VU, pink; EN, Oxford blue; CR, purple; EW, dark blue).

**Figure 3.**
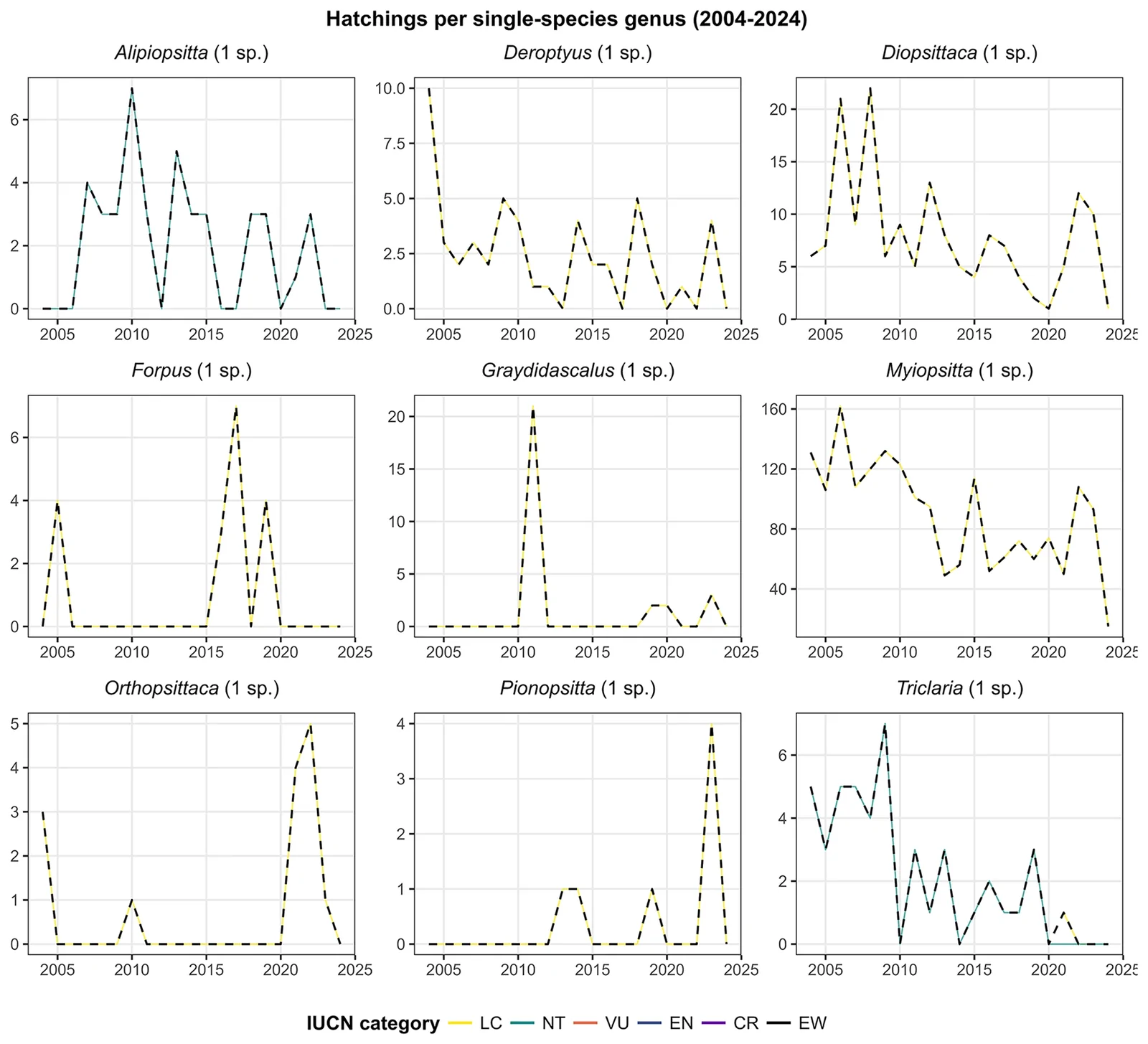
Number of hatchings per single-species non-threatened genus, 2004–2024. Each panel shows one genus with a single species; the solid and dashed lines coincide (dashed black line indicates the genus mean). Nine genera are shown. Most genera had low and irregular reproductive output, with *Myiopsitta* showing the highest and most consistent number of hatchings. Colours indicate IUCN categories (LC, yellow; NT, midnight blue; VU, pink; EN, Oxford blue; CR, purple; EW, dark blue).

Differences among IUCN threat categories were not significant. The estimate for EW species was highly uncertain and was therefore not interpreted. Overall, the model provided no evidence of differences in hatching rates among IUCN threat categories after accounting for temporal variation and total population size (table 2).

**Table 2.** Coefficients from a negative binomial mixed-effects model (GLMM) with linear parameterization (NB1) for Brazilian parrot hatchings in zoological institutions (2004–2024). (Notes: the model included standardized year (Year_c) and IUCN threat category as fixed effects, with total population size (total) included as an offset and an AR(1) temporal correlation structure within species. The intercept corresponds to Critically Endangered (CR) species at the mean year (2014; Year_c = 0) and total = 1. Year_c represents annual variation relative to 2014. Incidence rate ratios (IRRs) indicate multiplicative changes in the expected birth rate per unit increase in the corresponding predictor. Values are presented with 95% confidence intervals (CIs). Significant effects (*p* < 0.05) are shown in bold).

effect	estimate	s.e.	*z*-value	*p*-value	IRR	95% CI IRR
intercept (CR)	−2.875	0.331	−8.680	**<0.001**	0.056	0.029–0.108
Year_c	−0.222	0.045	−4.965	**<0.001**	0.801	0.734–0.874
IUCN EN	−0.019	0.307	−0.063	0.950	0.981	0.538–1.790
IUCN EW	−16.485	2169.415	−0.008	0.994	approx. 0.000	approx. 0–∞
IUCN LC	−0.340	0.335	−1.014	0.311	0.712	0.369–1.373
IUCN NT	0.028	0.364	0.077	0.939	1.028	0.504–2.100
IUCN VU	0.099	0.337	0.294	0.769	1.104	0.570–2.137

*Per capita* NetChange (NetChange_pc) varied substantially among species ( figure 4). Mean NetChange_pc values ranged from −0.236 in the cobalt-winged parakeet (*Brotogeris cyanoptera*) to 0.667 in the fiery-shouldered parakeet (*Pyrrhura egregia*), while the median ranged between −0.083 and 1.0, reflecting variation in both the direction and magnitude of population change. Overall, most species had mean values close to zero, suggesting broadly stable captive populations, although positive trends were observed in species such as the crimson-bellied parakeet (*Pyrrhura perlata coerulescens*) and the fiery-shouldered parakeet, while negative trends were observed in the cobalt-winged parakeet and the red-spectacled Amazon parrot (*Amazona pretrei*).

**Figure 4.**
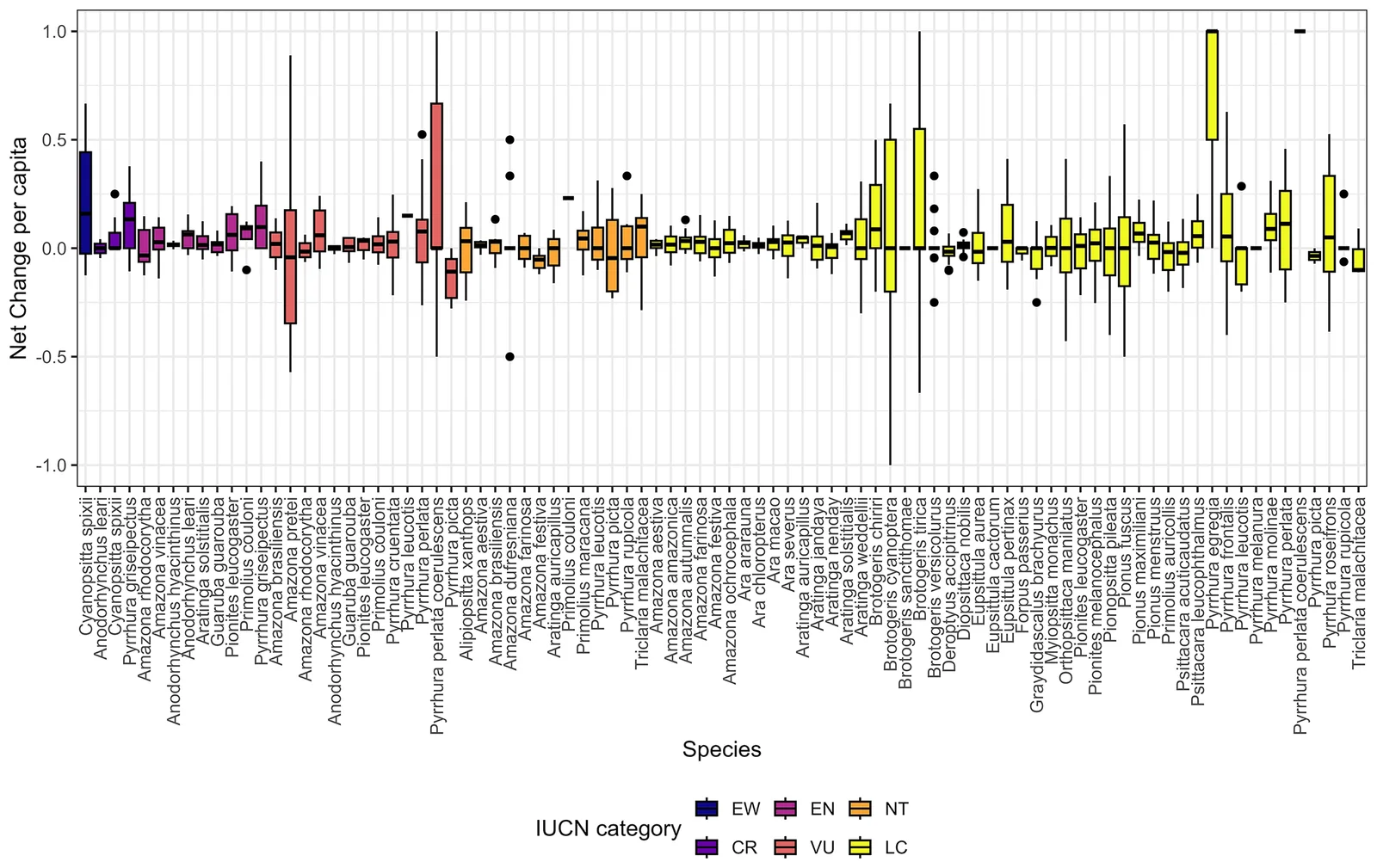
NetChange (NetChange_pc) distribution *per capita* for Brazilian parrot species in the study. Each box represents the interquartile range (25–75%) of the annual *per capita* population variation, the horizontal line indicates the median, and the whiskers extend to 1.5 × the interquartile range. Extreme values outside this range were removed to improve visualization. Boxplot colours indicate threat category by species according to the IUCN Red List. Species are grouped on the *x*-axis by their conservation status (Least Concern (LC, yellow); Near Threatened (NT, orange); Vulnerable (VU, red); Endangered (EN, pink); Critically Endangered (CR, purple); and Extinct in the Wild (EW, blue)).

Population rates varied widely among parrot species across all demographic metrics, including hatchings, mortality, acquisitions and net population change. Mean hatching rates ranged from 0.00 ± 0.00% in the cobalt-winged parakeet and in the cactus parakeet (*Eupsittula cactorum*) to 128.57 ± 197.60% in the crimson-bellied parakeet. Mean mortality rates varied between 0.00 ± 0.00% in the fiery-shouldered parakeet and 38.56 ± 96.02% in the plain parakeet (*Brotogeris tirica*), while mean acquisition rates ranged from 0.71 ± 3.19% in the short-tailed parrot (*Graydidascalus brachyurus*) to 194.23 ± 650.34% in the plain parakeet.

Net population declined in some species, such as the cobalt-winged parakeet (−23.58 ± 106.32%) and the painted parakeet (*Pyrrhura picta*: −8.48 ± 37.93%), while it was positive in others, such as the crimson-bellied parakeet (52.08 ± 60.05%) and the green-cheeked parakeet (*Pyrrhura molinae*) (13.69 ± 23.10%). Full species-level summaries are presented in the supplementary material, S3.

Across taxa, increases in population size were not primarily driven by hatchings, which were generally low, but by acquisitions. In most genera, acquisition rates (blue lines) showed higher peaks than hatching rates (green lines), and in several genera (e.g. *Cyanopsitta*, *Pyrrhura* and *Primolius*), acquisition rate spikes coincided with positive NetChange_pc values. Mortality rates frequently exceeded hatching rates, highlighting the contribution of acquisitions to population maintenance or increase. Genera with regular hatching rates (median ≥ 5%) showed consistent temporal variation across all demographic rates, with mortality frequently matching or exceeding hatching (figure 5).

**Figure 5.**
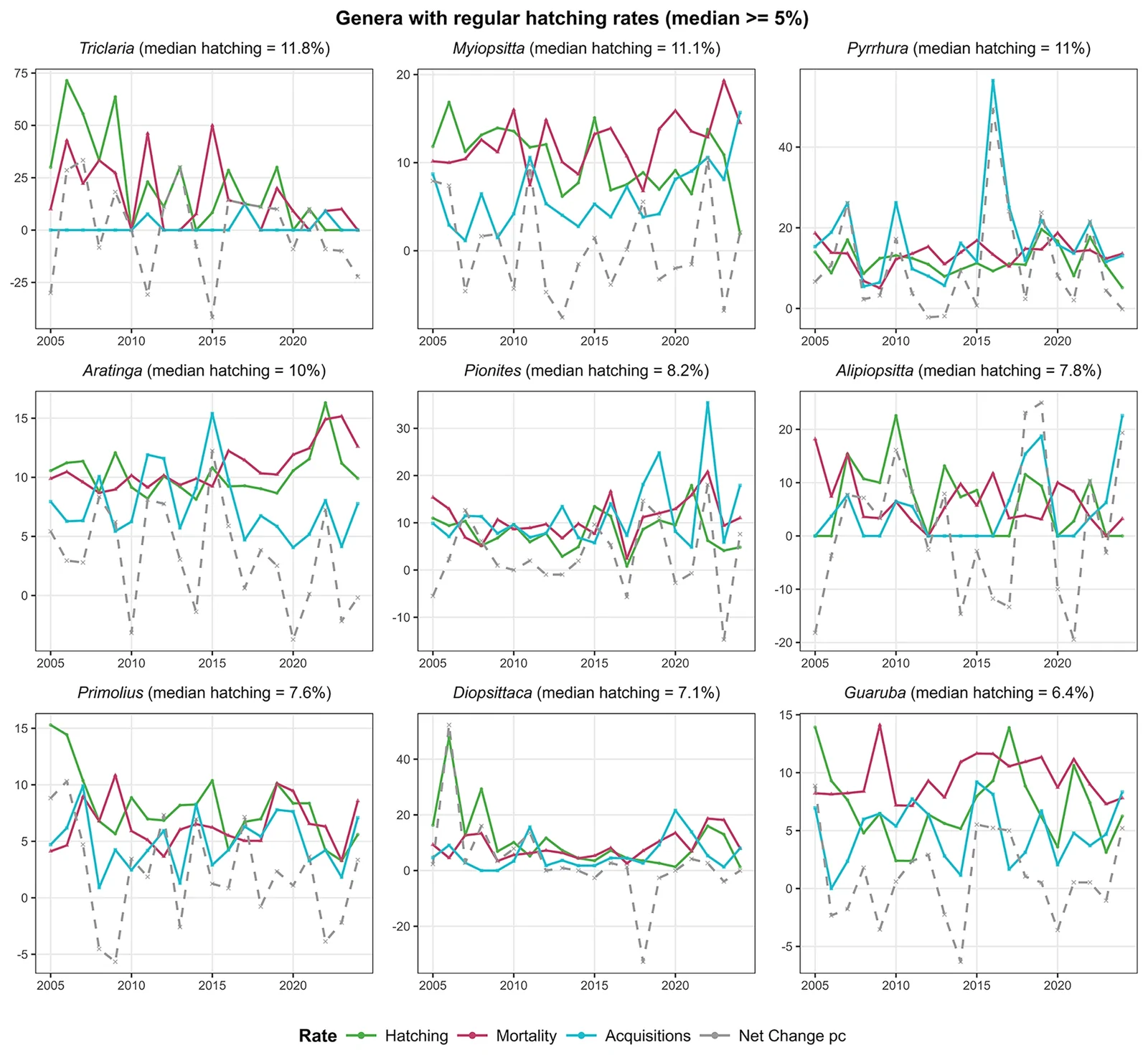
Genera with regular hatching rates in Brazilian parrot species maintained under human care, 2004–2024. Each panel represents a genus, with coloured lines indicating demographic rates: green, hatching rate; magenta, mortality rate; blue, acquisition rate; grey (dashed), *per capita* net population change (NetChange_pc). Values are expressed as percentages relative to the total population in the previous year. The nine genera shown had median hatching rates ≥ 5%, indicating consistent reproductive output across the study period. Among these, *Triclaria* (median hatching = 11.8%), *Myiopsitta* (11.1%) and *Pyrrhura* (11.0%) showed the highest median hatching rates. In most genera, mortality rates were similar to or exceeded hatching rates, and NetChange_pc values fluctuated around zero, indicating demographically stable rather than growing populations.

Genera with sporadic hatching rates (median 2–5%) showed isolated reproductive peaks separated by extended periods of near-zero hatching, with acquisition events playing a key role in population maintenance (figure 6). Genera with near-zero hatching rates (median < 2%) were characterized by reproduction that was effectively absent for most years, and population maintenance depended almost entirely on acquisitions (figure 7).

**Figure 6.**
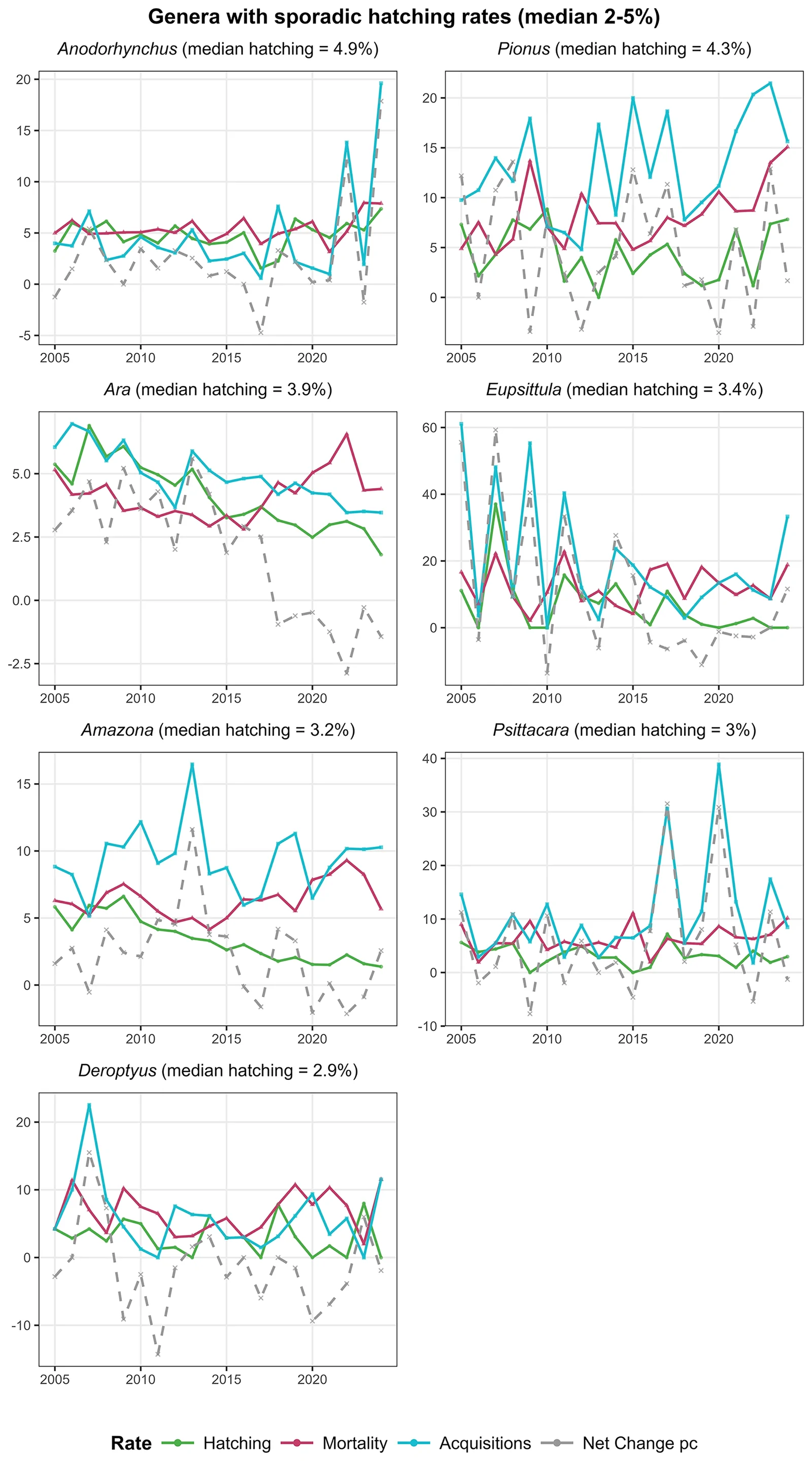
Genera with sporadic hatching rates in Brazilian parrot species maintained under human care, 2004–2024. Each panel represents a genus, with coloured lines indicating demographic rates as in figure 5 (green, hatching; magenta, mortality; blue, acquisitions; grey dashed, NetChange_pc). The seven genera shown had median hatching rates between 2 and 5%, indicating irregular reproductive output. In these genera, hatching occurred in isolated peaks separated by extended periods of near-zero reproduction, while mortality rates remained relatively stable. Notably, *Anodorhynchus* (median hatching = 4.9%) and *Pionus* (4.3%) showed the highest median hatching rates within this group, whereas *Ara* (3.9%) and *Amazona* (3.2%) showed more variable patterns.

**Figure 7.**
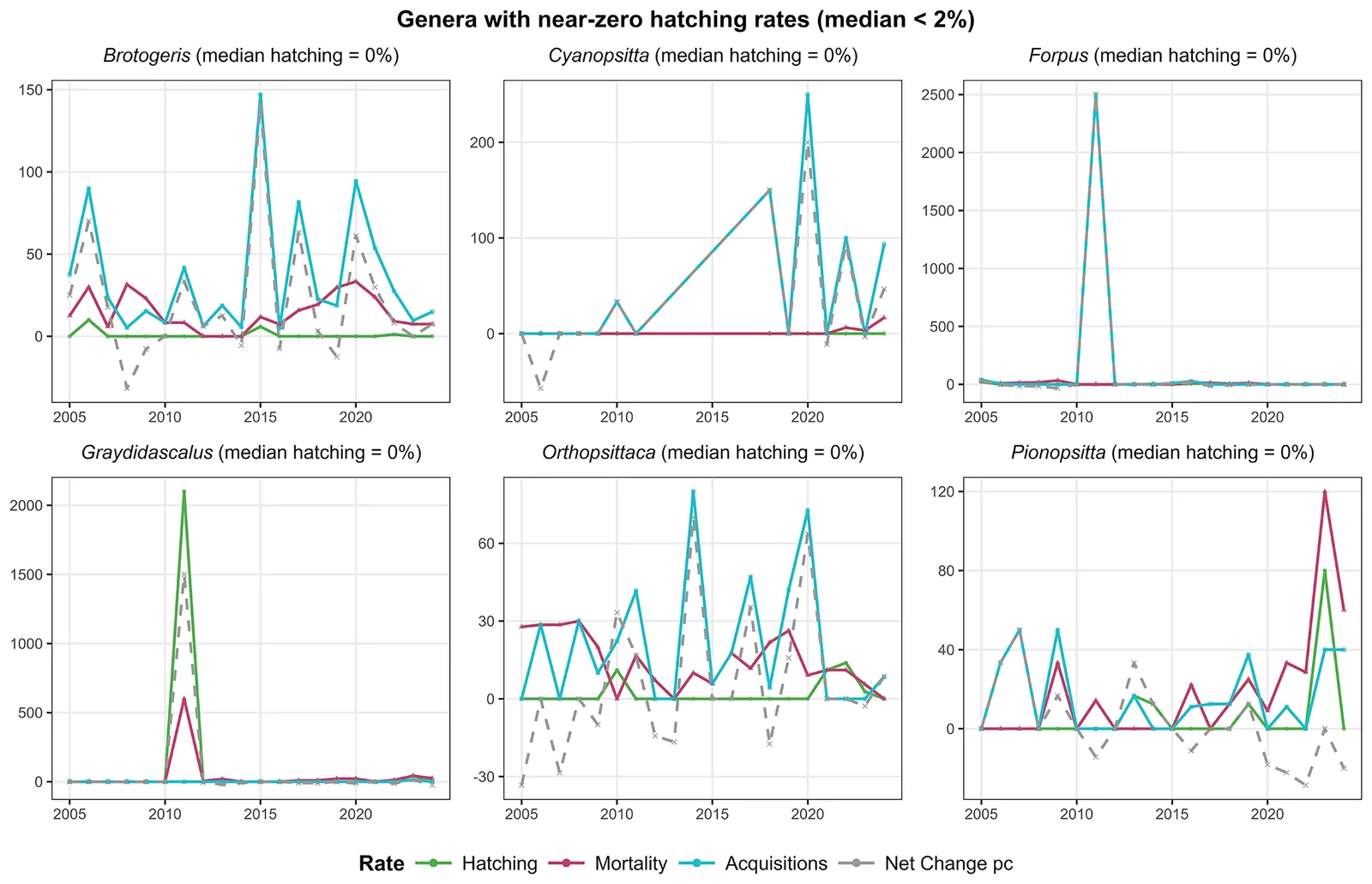
Genera with near-zero hatching rates in Brazilian parrot species maintained under human care, 2004–2024. Each panel represents a genus, with coloured lines indicating demographic rates as in figure 5 (green, hatching; magenta, mortality; blue, acquisitions; grey dashed, NetChange_pc). The six genera shown had median hatching rates of 0%, indicating reproduction that was effectively absent for most of the study period, despite occasional isolated peaks in some genera (e.g. *Forpus*, *Graydidascalus* and *Pionopsitta*).

To further assess temporal dynamics in population performance, species-specific trajectories of *per capita* net population change (NetChange_pc) were modelled over 2004–2024 using GAMs. Model assumptions were assessed using the procedures described in §3.5. ‘Model diagnostics’. The global GAMs of NetChange_pc did not meet diagnostic assumptions for any specification (supplementary material, S2, tables S2–S7), reflecting the intrinsic heterogeneity of this metric across species; these models are therefore reported for descriptive purposes only, and inference is based on genus-level models. For most species, the smooth term for year was not significant (all *p* > 0.05), indicating no consistent temporal trends in NetChange_pc.

Species-specific GAMs were fitted for 59 species, of which four were excluded owing to insufficient data (*N* < 5 or < 5 unique values of NetChange_pc; supplementary material, S2, table S12). For most species (*n* = 46), the smooth term for year was not significant (*p* > 0.05), indicating no consistent temporal trends in NetChange_pc. Significant temporal trends (*p* < 0.05) were detected in 13 species, all of which showed declining trajectories. The strongest signals were observed in the genus *Ara*: the scarlet macaw (*Ara macao*; effective degrees of freedom (EDF) = 2.770, *χ*^2^ = 33.187, *p* < 0.001; adjusted *R*^2^ = 0.615; deviance explained = 66.9%), the blue-and-yellow macaw (*Ara ararauna*; EDF = 2.302, *χ*^2^ = 20.014, *p* < 0.001; adjusted *R*^2^ = 0.496) and the red-and-green macaw (*Ara chloropterus*; EDF = 3.528, *χ*^2^ = 16.994, *p* = 0.002; adjusted *R*^2^ = 0.457). Additional declining trends were detected in the golden-collared macaw (*Primolius auricollis*; *p* < 0.001; *R*^2^ = 0.358), the pileated parrot (*Pionopsitta pileata*; *p* = 0.002; *R*^2^ = 0.333), the orange-winged Amazon parrot (*Amazona amazonica*; *p* = 0.021; *R*^2^ = 0.179), the nanday parakeet (*Aratinga nenday*; *p* = 0.021; *R*^2^ = 0.177), the sun conure (*Aratinga solstitialis*; *p* = 0.022; *R*^2^ = 0.176), the hyacinth macaw (*Anodorhynchus hyacinthinus*; *p* = 0.029; *R*^2^ = −0.092), the dusky-headed parakeet (*Aratinga weddellii*; *p* = 0.043; *R*^2^ = 0.255), the brown-throated parakeet (*Eupsittula pertinax*; *p* = 0.047; *R*^2^ = 0.209), the short-tailed parrot (*Gr. brachyurus*; *p* = 0.049; *R*^2^ = 0.003) and the blue-headed macaw (*Primolius couloni*; *p* = 0.049; *R*^2^ = 0.124); a marginal declining trend was detected in the grey-breasted parakeet (*Pyrrhura griseipectus*; *p* = 0.090). Notably, the Spix's macaw (*Cyanopsitta spixii*, EW) showed a positive but non-significant trend (*p* = 0.237; adjusted *R*^2^ = 0.072), with fitted values increasing from −0.014 in 2004 to +0.274 in 2024, and the golden parakeet (*Guaruba guarouba*, VU) showed no detectable temporal trend (*p* = 0.745). Although models were fitted at the species level, figures 8–10 show genus-level aggregates to facilitate comparison of broader taxonomic patterns in NetChange_pc trajectories. Model assumptions were assessed as described in §3.5. ‘Model diagnostics’; all 59 species-specific GAMs met DHARMa residual uniformity assumptions (*p* > 0.05; supplementary material, S2, table S12), indicating that model assumptions were adequately met.

**Figure 8.**
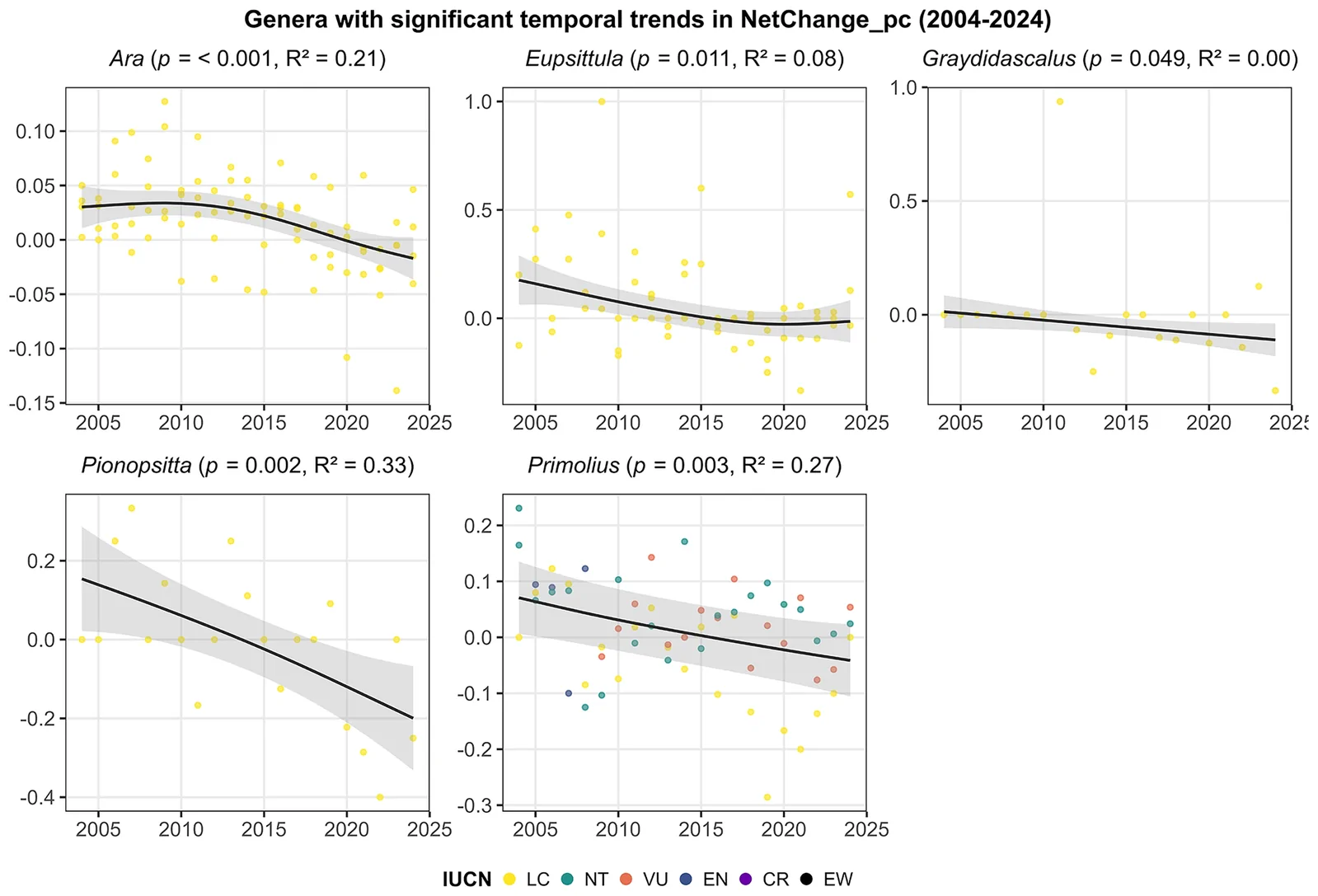
Genera with significant temporal trends in NetChange_pc, 2004–2024. Points are observed values; lines are GAM-fitted trends; shaded areas are 95% confidence intervals. Five genera showed significant trends (*p* < 0.05), most of them negative, indicating declining captive populations. Colours indicate IUCN categories (LC, yellow; NT, midnight blue; VU, pink; EN, Oxford blue; CR, purple; EW, dark blue).

**Figure 9.**
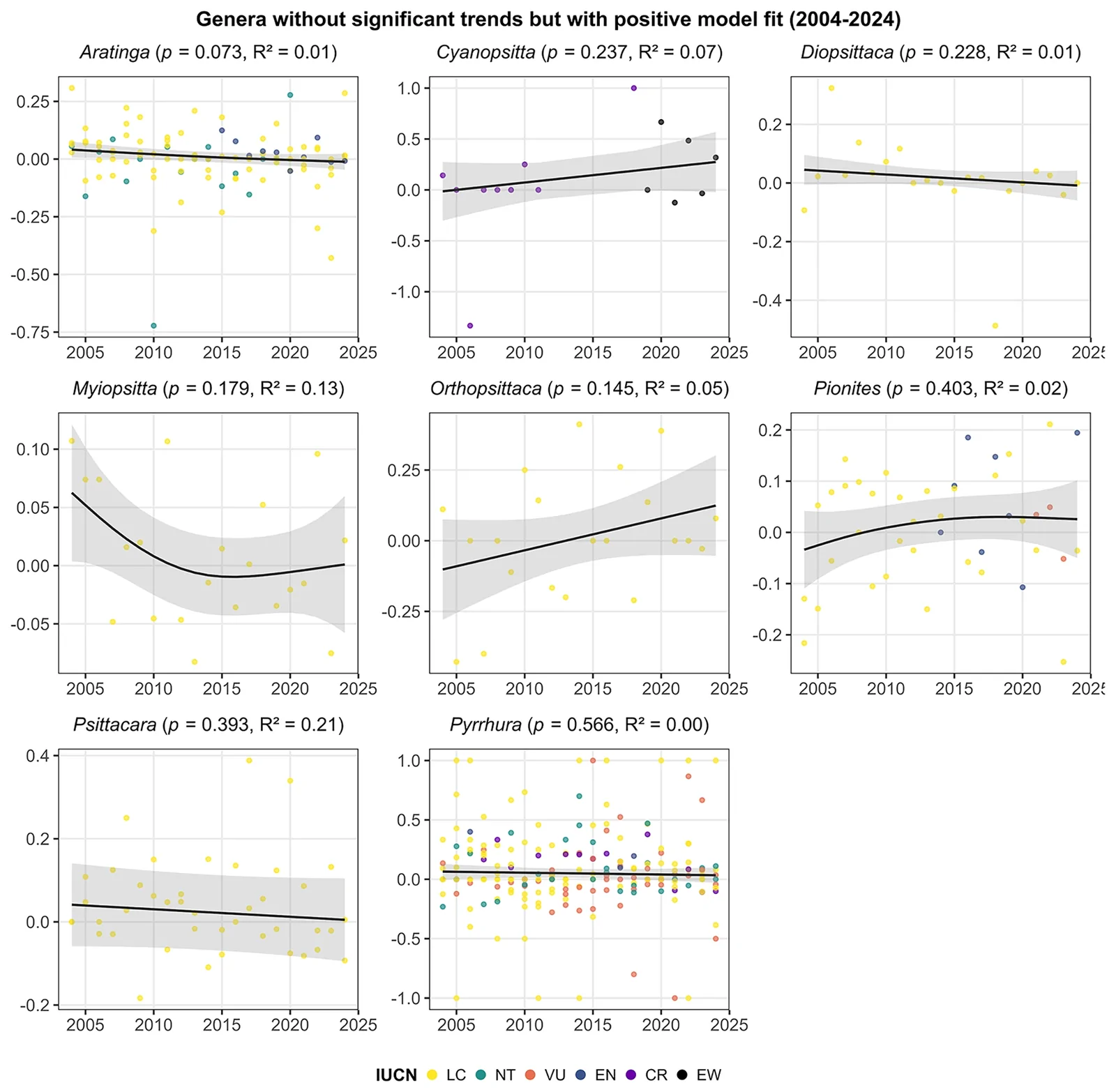
Genera without significant trends but with positive model fit, 2004–2024. Points are observed values; lines are GAM-fitted trends; shaded areas are 95% confidence intervals. Eight genera showed no significant trend (*p* > 0.05) but positive adjusted *R*^2^, indicating relatively stable populations. *Cyanopsitta* (Spix's macaw, EW) showed a positive, non-significant trend. Colours indicate IUCN categories (LC, yellow; NT, midnight blue; VU, pink; EN, Oxford blue; CR, purple; EW, dark blue).

**Figure 10.**
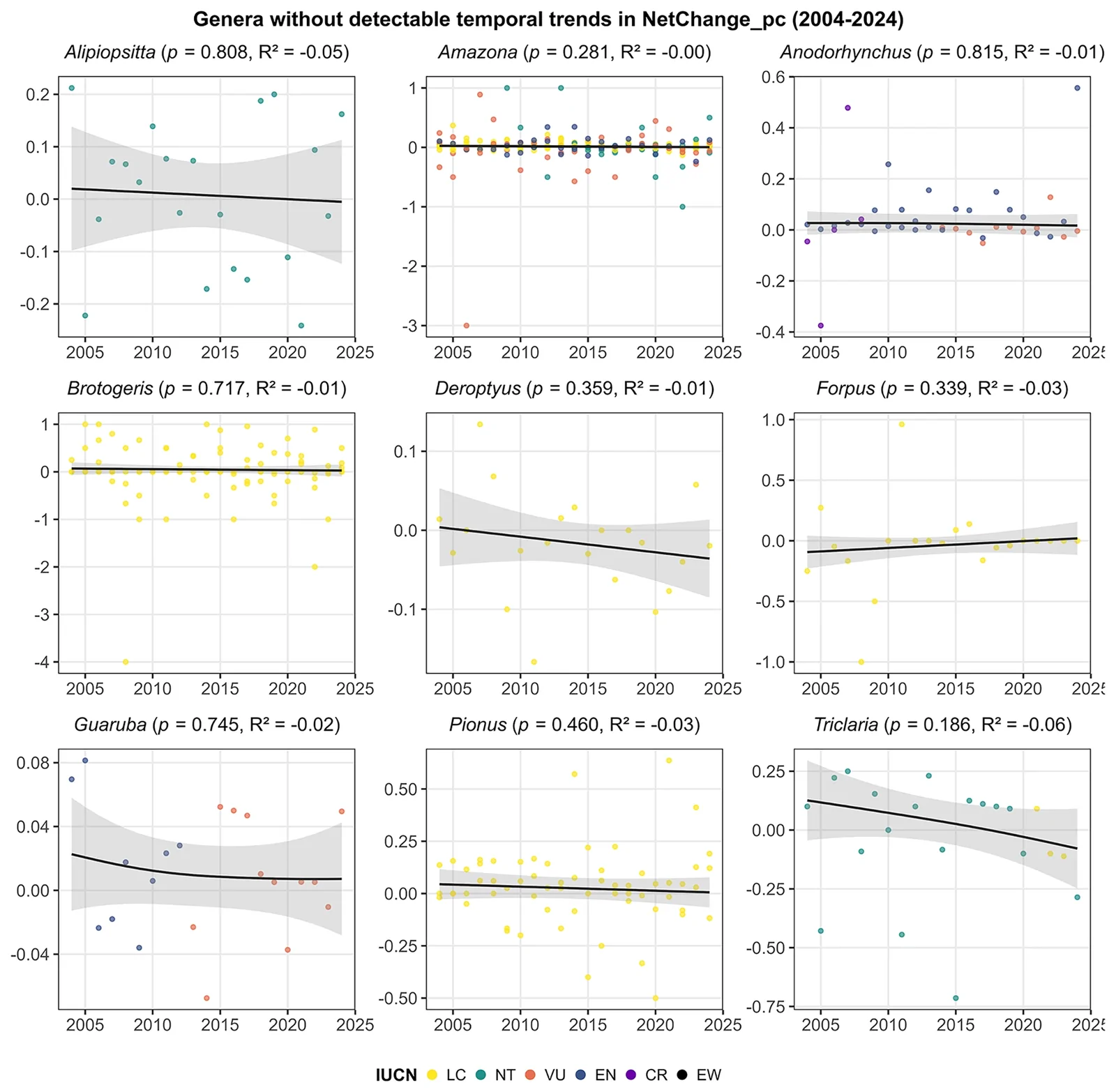
Genera without detectable temporal trends, 2004–2024. Points are observed values; lines are GAM-fitted trends; shaded areas are 95% confidence intervals. Nine genera showed no significant trend (*p* > 0.05) and non-positive adjusted *R*^2^, indicating the absence of a detectable temporal signal rather than model failure. Colours indicate IUCN categories (LC, yellow; NT, midnight blue; VU, pink; EN, Oxford blue; CR, purple; EW, dark blue).

### 4.2. Conservation status and demographic performance (H2)

Reproductive output was assessed using a negative binomial GLMM based on 1259 species-year observations from 63 species, with annual hatchings modelled relative to captive population size and year, and species included as a random effect. The model had adequate diagnostics and explained 28.8% of the variance through fixed effects and 37.8%, including the species-level random effect (marginal *R*^2^ = 0.288; conditional *R*^2^ = 0.378). Reproductive output declined over time, with lower hatching rates relative to captive population size (*β* = −0.224 ± 0.019 s.e., *z* = −11.79, *p* < 0.001). IUCN threat category also had an overall effect ($\chi_{5}^{2}$ = 21.820, *p* = 0.0006). Tukey-adjusted estimated marginal means indicated higher predicted hatching rates for EN than for LC species (*p* = 0.0003) and for VU than for LC species (*p* = 0.0013). Other pairwise comparisons were not significant (*p* > 0.69), including comparisons involving CR species. The estimate for EW species was highly uncertain, reflecting the limited information available for this category.

For population growth, the global model of *per capita* population change was not used for formal inference because it did not meet the predefined diagnostic criteria. We therefore assessed the relationship between IUCN status and NetChange_pc using genus-level GAMs. Nine genus-level models converged successfully, of which five, *Aratinga*, *Pionites*, *Primolius*, *Pyrrhura* and *Triclaria*, met all predefined diagnostic criteria for residual uniformity, dispersion, outliers and quantile deviations. Among these five models, explanatory power was generally low, with adjusted *R*^2^ values ranging from −0.112 to 0.282 and deviance explained ranging from 3.4 to 31.5%, indicating limited ability to account for variation in *per capita* population change. None of the five models showed a significant overall effect of IUCN threat category (*Aratinga*: *F*_2,100_ = 1.194, *p* = 0.307; *Pionites*: *F*_2,37.74_ = 0.619, *p* = 0.544; *Primolius*: *F*_3,57.35_ = 1.085, *p* = 0.363; *Pyrrhura*: *F*_4,200.15_ = 0.815, *p* = 0.517; *Triclaria*: *F*_1,17.84_ = 0.028, *p* = 0.869). Thus, within the genus-level models that met the diagnostic criteria, there was no evidence of systematic differences in *per capita* population change among IUCN threat categories.

### 4.3. Sex ratio and population stability (H3)

A GAMM was fitted to examine the relationship between *per capita* net population change (NetChange_pc), sex ratio and IUCN threat status. Overall model fit was low (adjusted *R*^2^ = 0.013; deviance explained = 1.98%), and no significant effects of IUCN threat status were detected (all *p* > 0.15). By contrast, sex ratio was nonlinearly associated with NetChange_pc (EDF = 3.31, *F* = 4.26, *p* = 0.011). NetChange_pc increased at low to moderate sex ratios (0–0.45), peaked around 0.45 and then declined between approximately 0.45 and 0.75. At higher sex ratios (0.75–1), NetChange_pc increased again, although observations in this range were limited (figure 11). Thus, the relationship between sex ratio and *per capita* population change was nonlinear, with the highest predicted values occurring around a sex ratio of 0.45, while estimates at higher sex ratios were less well supported by the available observations.

**Figure 11.**
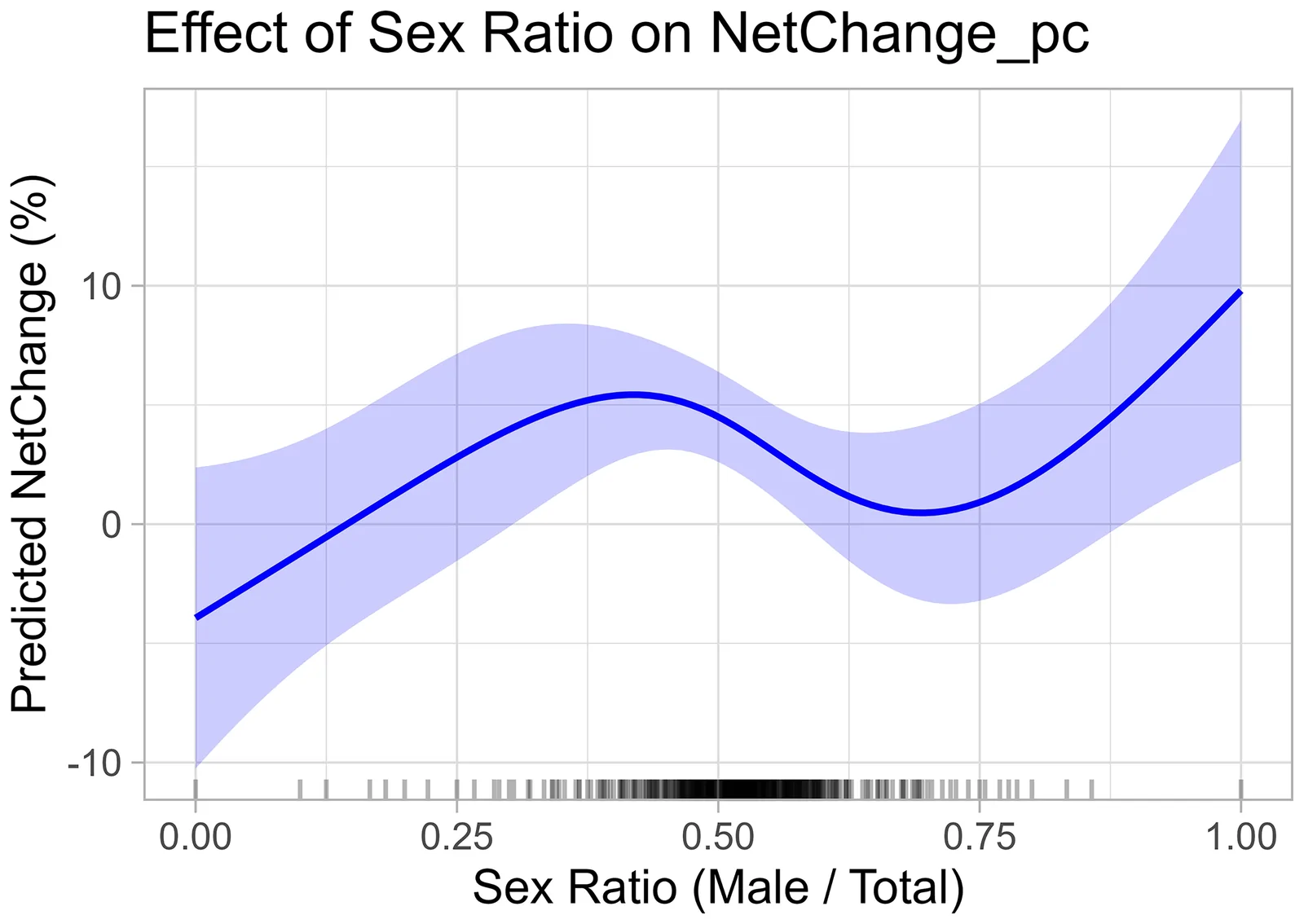
Predicted effect of sex ratio on *per capita* population change (NetChange_pc) in captive Brazilian parrots, 2004–2024. The solid line shows the GAMM-fitted relationship between sex ratio (SexRatio = males/total individuals) and predicted NetChange_pc, with the shaded area indicating the 95% confidence interval. Tick marks along the *x*-axis show the distribution of observations across the sex-ratio range. The fitted curve indicates a nonlinear relationship, with predicted NetChange_pc increasing at low to moderate sex ratios, peaking at approximately 0.45, and then declining between approximately 0.45 and 0.75. Predicted NetChange_pc increased again at higher sex ratios, although observations in this range were limited.

### 4.4. Trade pressure and extinction risk (H4)

The GAMM explained 45.5% of the variance (adjusted *R*^2^ = 0.455). Recorded trade volume was associated with threat status, captive population size, importing and exporting regions, declared purposes of use, specimen origin, and year.

Captive population size had a positive effect on trade volume (log_captive: estimate = 0.08846, s.e. = 0.03117, *t* = 2.838, *p* = 0.004545), indicating that species with larger captive populations were traded more frequently. Threat level was positively associated with trade volume, with species at higher extinction risk appearing more frequently in trade records (estimate = 0.05635, s.e. = 0.02521, *t* = 2.235, *p* = 0.025). Trade patterns varied across regions, with Asia showing the strongest positive association with import volume (estimate = 1.24640, s.e. = 0.04198, *t* = 29.694, *p* < 0.001), followed by Europe (estimate = 0.19374, s.e. = 0.04679, *t* = 4.141, *p* < 0.001) and the Americas (estimate = 0.16592, s.e. = 0.04931, *t* = 3.365, *p* < 0.001) with similar positive trends. Oceania showed a positive but non-significant effect (estimate = 0.32740, s.e. = 0.32321, *t* = 1.013, *p* = 0.311), probably reflecting the small number of records for this region. By contrast, all exporting regions showed significant negative effects relative to Africa (reference category): the Americas (estimate = −0.56323, s.e. = 0.04296, *t* = −13.110, *p* < 0.001), Asia (estimate = −0.94363, s.e. = 0.04159, *t* = −22.690, *p* < 0.001), Europe (estimate = −1.16765, s.e. = 0.04049, *t* = −28.842, *p* < 0.001) and Oceania (estimate = −1.77146, s.e. = 0.17098, *t* = −10.360, *p* < 0.001), indicating lower export volumes than from African sources (figure 12).

**Figure 12.**
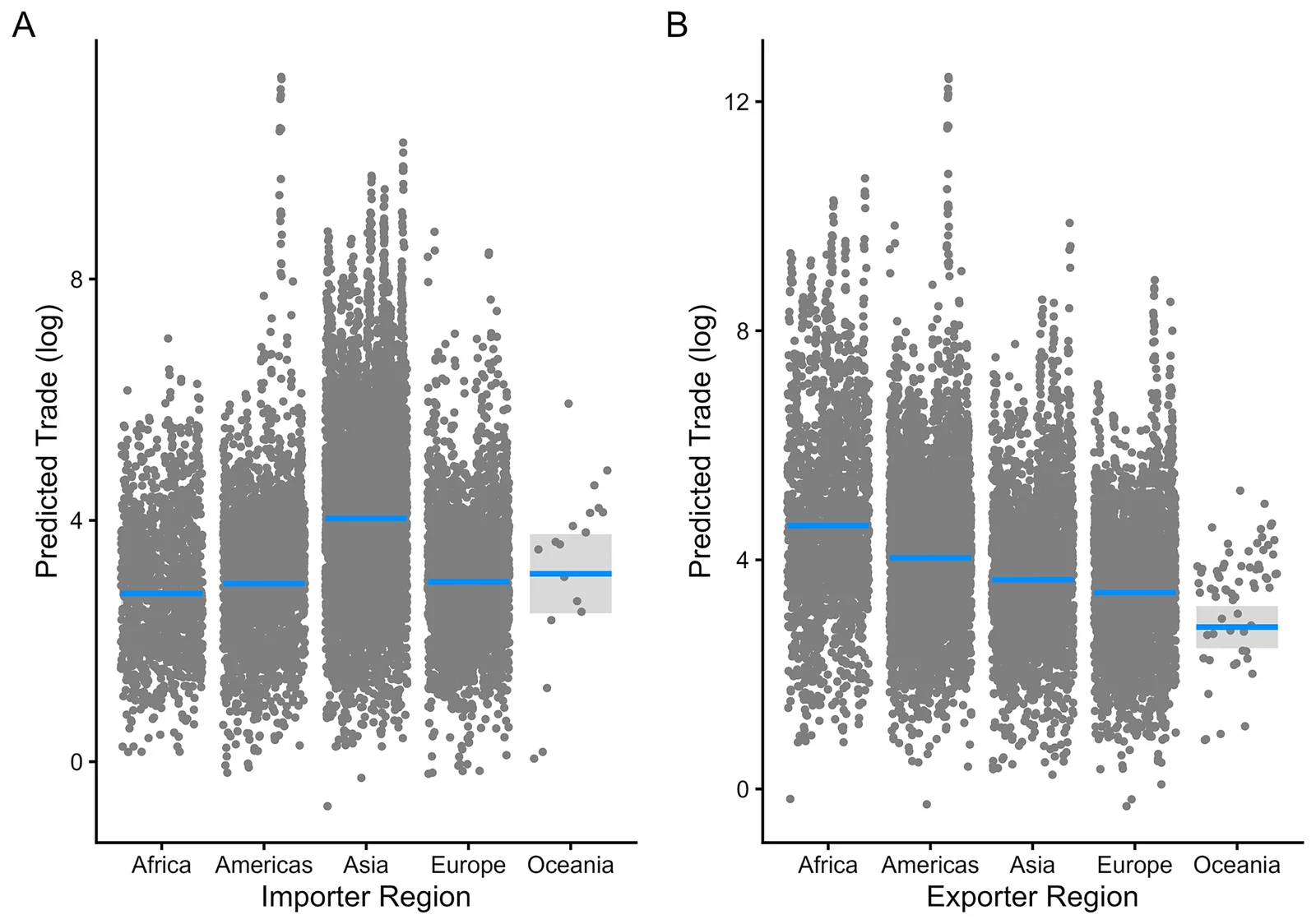
Predicted parrot trade by region. (A) Predicted trade by importing region. (B) Predicted trade by exporting region. Values were adjusted by the International Union for Conservation of Nature (IUCN) threat status, captive population size, trade purpose, specimen source and year using the GAMM model. Points represent the log-adjusted predictors for each region. The horizontal line indicates the mean model-adjusted trade.

Transaction purpose was also associated with trade volume. Commercial transactions (code T) had the strongest positive effect (estimate = 1.02881, s.e. = 0.04100, *t* = 25.095, *p* < 0.001), indicating that commercial trade accounted for the largest share of total trade. Reintroduction or introduction into the wild (code N) showed a positive but marginal effect (estimate = 0.50525, s.e. = 0.25636, *t* = 1.971, *p* = 0.0488), probably reflecting international transfers of individuals for conservation programmes rather than commercial trade. Several other purposes showed significant negative effects, most notably hunting trophies (code H: estimate = −2.946, *p* < 0.001), personal use (code P: estimate = −0.805, *p* < 0.001), scientific use (code S: estimate = −0.547, *p* = 0.008) and circus or travelling exhibitions (code Q: estimate = −0.541, *p* < 0.001). Zoo-related transactions also showed a significant negative effect (code Z: estimate = −0.320, *p* < 0.001). Medical research (code M) and law enforcement (code L) were not significant (*p* > 0.15) (figure 13).

**Figure 13.**
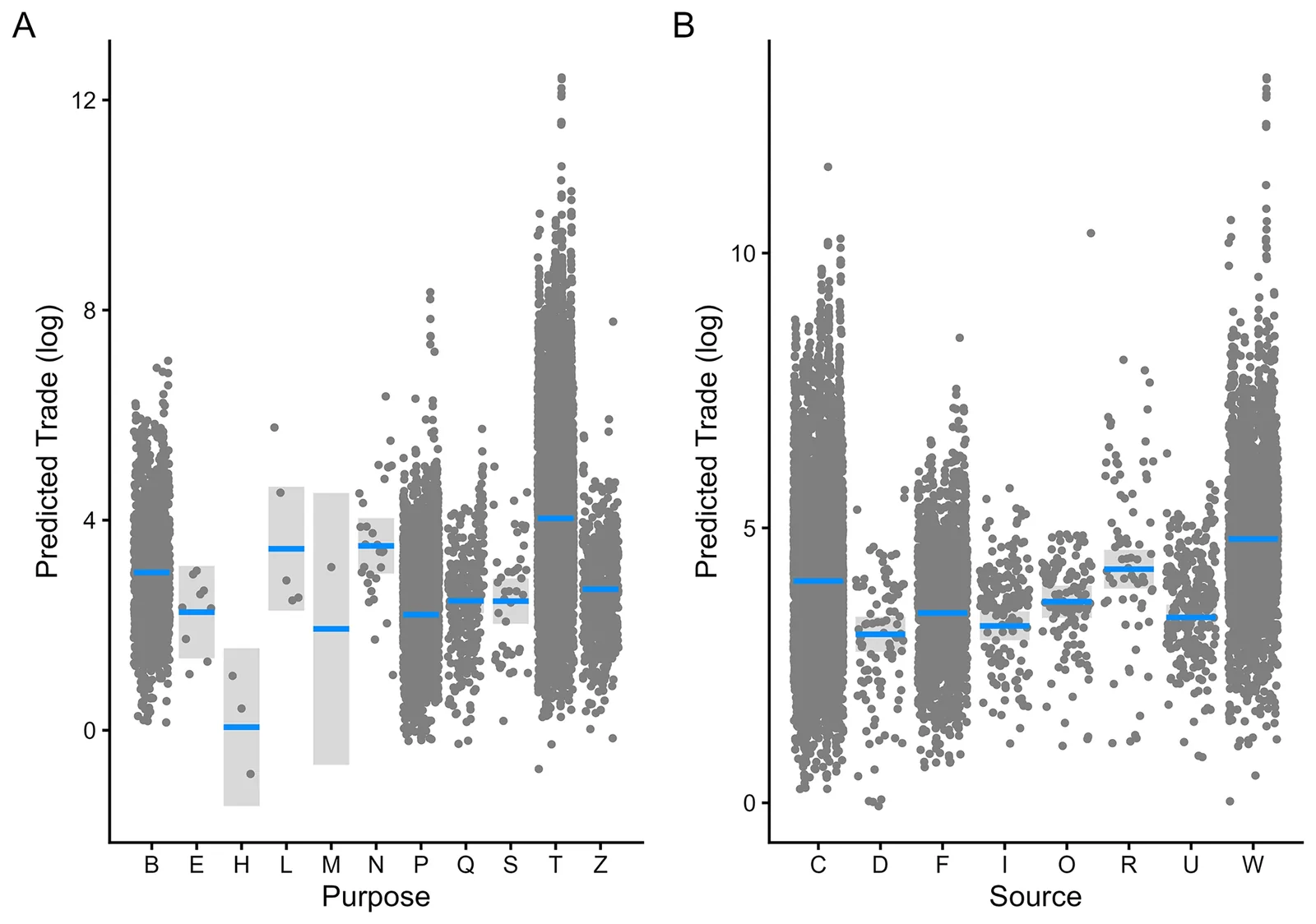
Effects of transaction purpose (A) and specimen source (B) on the predicted Psittacidae trade (log scale), adjusted using the GAMM model. Predicted values represent model-adjusted estimates with other variables held constant (captive population size, importing and exporting regions, year, and IUCN threat status). Transaction purpose codes: B, breeding in captivity or artificial propagation; E, educational; G, botanical garden; H, hunting trophy; L, law enforcement/judicial/forensic; M, medical (including biomedical research); N, reintroduction or introduction into the wild; P, personal; Q, circus or travelling exhibitions; S, scientific; T, commercial; Z, zoo. Source codes: A, artificially propagated plants; C, animals bred in captivity; D, appendix-I animals/plants bred in captivity for commercial purposes; F, animals born in captivity (F1+) that do not meet the definition of ‘bred in captivity’; I, confiscated or seized specimens; O, pre-convention specimens; R, ranched specimens; U, source unknown; W, taken from the wild; X, marine specimens not under national jurisdiction; Y, plants meeting the criteria for ‘assisted production’.

Specimen origin was also associated with trade volume. Wild-caught specimens (code W) showed a strong positive effect (estimate = 0.76507, s.e. = 0.03759, *t* = 20.351, *p* < 0.001), indicating that wild individuals continue to contribute substantially to the recorded trade in Brazilian parrot species. By contrast, several controlled or non-wild sources showed significant negative effects: captive-bred appendix I specimens (code D: estimate = −0.969, *p* < 0.001), confiscated or seized individuals (code I: estimate = −0.816, *p* < 0.001), specimens of unknown origin (code U: estimate = −0.661, *p* < 0.001), F1+ captive-born specimens (code F: estimate = −0.581, *p* < 0.001) and pre-convention specimens (code O: estimate = −0.376, *p* = 0.002). Ranched specimens (code R) were not significantly different from the reference (estimate = 0.216, *p* = 0.176) (figure 13).

Finally, the smooth term for year was highly significant (EDF = 10.05, *F* = 5.933, *p* < 0.001), indicating a nonlinear temporal pattern in trade volume characterized by alternating periods of increase and decline over the study period (figure 14).

**Figure 14.**
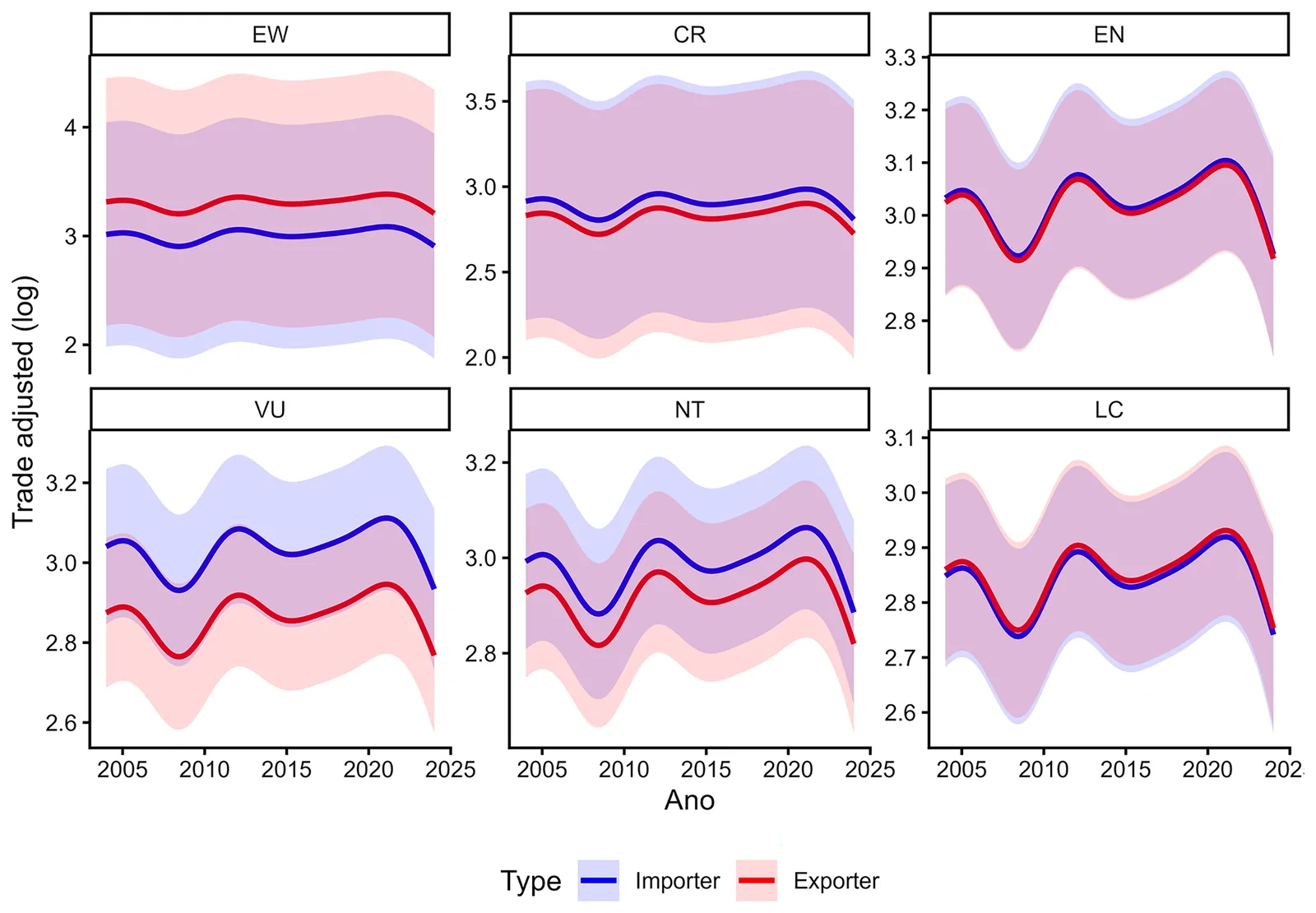
Predicted temporal trends in trade of Brazilian parrot species by the IUCN risk category and the trade type. Lines represent GAMM model predictions for each year, with import trade in blue and export trade in red. Shaded areas indicate the 95% confidence intervals. Each panel corresponds to an IUCN risk category: Least Concern (LC), Near Threatened (NT), Vulnerable (VU), Endangered (EN), Critically Endangered (CR), and Extinct in the Wild (EW).

## 5. Discussion

In this study, we assessed demographic performance and international trade patterns in *ex situ* populations of Brazilian parrot species by testing four hypotheses concerning demographic trends, threat status, sex ratio and trade. The results provide limited or partial support for some predictions and contradict others, highlighting the importance of considering effect magnitude, demographic context and management conditions when interpreting *ex situ* population performance. Despite the population trends observed over the 20-year period assessed in this study, these trends should not be interpreted in isolation as evidence of long-term population viability, as demographic trajectories may also reflect changes in population size, management practices and other conditions affecting *ex situ* populations.

Our first hypothesis predicted that zoo-managed populations would show positive demographic performance, with hatchings exceeding deaths and resulting in population growth under structured management and breeding programmes. This prediction was not consistently supported. Captive parrot populations did not show general demographic growth over the analysed period, and reproductive output declined over time, a pattern that may be relevant to population management. The year IRR was 0.80, corresponding to an approximately 20% reduction in the expected hatching rate for each one-unit increase in the centred year variable. If sustained over multiple years, even relatively modest reductions in reproductive output can progressively reduce captive populations’ capacity to replace losses and generate demographic surplus. This is consistent with species-level NetChange_pc trajectories, as most populations were stable or declining rather than consistently increasing. At the same time, absolute reproductive output remained low. At the mean year of the study, predicted hatching rates ranged from approximately 0.04 to 0.06 per individual across the main IUCN categories, indicating that differences among categories were relatively small in absolute terms. Thus, the conservation significance of the temporal decline lies primarily in its potential cumulative effect on population replacement rather than in evidence of abrupt demographic collapse.

The distinction between population maintenance and demographic growth is particularly important when interpreting these results. For captive parrot populations, long-term persistence may depend not only on reproduction but also on coordinated management, including external acquisitions, transfers and other forms of demographic supplementation [8,48,49]. Importantly, acquisitions recorded in ZIMS should not necessarily be interpreted as representing additions of new founders to the overall *ex situ* population, because many may reflect transfers of individuals already maintained by another zoological institution [50]. Acquisition records should therefore be interpreted primarily as indicators of population supplementation or redistribution within the *ex situ* network rather than as direct evidence of demographic growth at the global population level. Evidence from other *ex situ* programmes supports this distinction. For example, the captive Asian elephant (*Elephas maximus*) population in Myanmar was maintained at a relatively large size for decades but was estimated to be demographically unsustainable because low birth rates and high mortality required continued supplementation from wild populations [51]. Similarly, population viability analyses of captive Columbia Basin pygmy rabbits (*Brachylagus idahoensis*) showed that population persistence did not necessarily provide sufficient surplus production to support sustained removals for reintroduction and supplementation, with juvenile survival and recruitment strongly influencing population growth [52]. These examples illustrate that maintaining population size and generating demographic growth or a harvestable surplus are distinct outcomes. A population can consequently remain numerically stable while lacking demographic autonomy, particularly when stability is maintained through demographic supplementation rather than reproduction within the population [51–53]. In this context, near-zero NetChange_pc values may indicate that an insurance population is being maintained, but they do not necessarily demonstrate demographic self-sustainability or sufficient surplus production for future population expansion, reintroduction or reinforcement programmes. Evidence from captive white rhinoceroses further illustrates this distinction, as population viability depended on reproductive performance that varied substantially among institutions, with the European population projected to decline under current demographic parameters despite its continued maintenance within the *ex situ* network [54]. Conversely, positive NetChange_pc values associated with acquisitions may reflect redistribution among institutions rather than intrinsic population growth, particularly when individuals are transferred within the same managed network. Consequently, local changes in institutional population size should be distinguished from demographic growth generated through reproduction.

The limited demographic growth observed across many species may partly reflect parrots’ life-history characteristics. Many species exhibit delayed sexual maturity, low annual reproductive output and strong dependence on specific nesting cavities, which are often scarce or contested resources in natural systems [55]. Such traits can constrain demographic growth both in the wild and under human care, particularly in low-fecundity species with longer breeding intervals [20,56–59]. The species included in this comparative analysis also differ substantially in life-history traits, reproductive biology, generation length, social organization and husbandry requirements [60–62]. Consequently, demographic rates and population trajectories in zoological institutions are likely to reflect species-specific biological constraints as well as differences in management conditions [13,63]. Although the models accounted for repeated observations and between-species heterogeneity, the available ZIMS data did not allow direct evaluation of these species-specific biological and management factors.

Despite these constraints, zoological institutions may function as demographic refuges by maintaining individuals over extended periods and contributing to genetic persistence through assurance populations that buffer stochastic extinction risks and preserve genetic diversity [50,64]. However, the generally low or negative NetChange_pc values observed in this study suggest that these populations more often function as stabilizing insurance populations than as self-sustaining systems capable of generating demographic surplus. This distinction matters for conservation management because numerical stability may depend on ongoing institutional coordination and therefore does not necessarily indicate sufficient intrinsic reproductive capacity to support population expansion or other conservation applications [65,66].

Because ZIMS aggregates records from zoological institutions operating under different management conditions, additional unexplained variation may arise from differences in husbandry practices, breeding-management strategies, enclosure capacity, social grouping, pairing decisions, institutional breeding priorities and population-management objectives. Such factors can have direct demographic consequences. For example, space availability can constrain how many endangered bird institutions can maintain and propagate, limiting opportunities for population growth independent of the species’ intrinsic reproductive capacity [67]. Similarly, breeding outcomes may depend on managed pairing decisions rather than on reproductive biology alone. Martin-Wintle *et al.* [68] showed that incorporating mate choice into *ex situ* breeding programmes can improve reproductive performance and increase the efficiency of population management, highlighting how institutional breeding decisions may influence demographic outcomes. Consequently, observed low reproductive output in zoological populations should not necessarily be interpreted as evidence of biological limitation; it may also reflect deliberate management decisions, space constraints, pair incompatibility or population-management strategies that prioritize demographic stability, genetic diversity or other conservation objectives over numerical expansion [65,67,68]. This consideration is particularly relevant when interpreting interspecific differences in demographic performance, because species represented predominantly in institutions with different breeding capacities or management objectives may exhibit systematically different demographic trajectories. Conversely, differences in institutional participation and reporting completeness could affect the apparent frequency of hatchings, mortality, acquisitions or transfers. Because the dataset did not include institution-level management variables or measures of reporting effort, these sources of heterogeneity could not be incorporated directly into the models and may account for some of the unexplained variation in demographic performance.

These sources of variation are particularly relevant when interpreting reproductive patterns because reproductive success in captivity is also shaped by animal welfare and the quality of the captive environment [69–71]. For example, variation in husbandry and enclosure conditions among zoological institutions has been associated with differences in behaviour and breeding performance in captive penguins, with colony structure and breeding management contributing to reproductive outcomes [70]. Similarly, environmental enrichment has been shown to influence reproductive performance in captive animals, indicating that aspects of the captive environment can affect reproduction beyond the intrinsic biological characteristics of the species [72]. More broadly, welfare-oriented management is considered an important component of conservation breeding because environmental, behavioural and social conditions can affect stress, health, survival and reproductive performance [69]. Frameworks developed to systematically assess enclosure quality for terrestrial animals in captivity have also highlighted the importance of enclosure conditions, emphasizing variation in the physical and behavioural features of captive environments among institutions [71]. These factors are unlikely to be uniform across zoological institutions; consequently, some interspecific and temporal variation observed in ZIMS may reflect differences in management practices as well as biological constraints. Even under structured management, behavioural and physiological constraints in captivity may limit reproductive output and contribute to the relatively low NetChange_pc values observed across many species [20,50,56]. Thus, statistically detectable changes in reproductive output should not necessarily be interpreted as evidence of biologically meaningful improvements in population viability, particularly when demographic gains remain small or are offset by mortality and other population losses.

Species-level patterns further illustrate the distinction between demographic maintenance and autonomous growth. The genus with the most negative mean NetChange_pc value was *Forpus* (−0.037), which persisted despite occasional hatching events and reliance on acquisitions. Similarly, genera with near-zero median hatching rates, such as *Brotogeris*, *Cyanopsitta*, *Graydidascalus*, *Orthopsittaca* and *Pionopsitta*, showed high interannual variability, with positive changes often associated with acquisitions or transfers rather than sustained breeding. These movements may have increased population size at particular institutions without necessarily increasing the overall *ex situ* population size or genetic diversity. Comparable challenges have been described in other conservation breeding programmes, where maintaining viable populations requires coordinated management across institutions rather than relying on reproduction within individual facilities alone [73]. For example, the Puerto Rican parrot (*Amazona vittata*) remained in a prolonged demographic bottleneck despite intensive conservation efforts, with population growth constrained by demographic, environmental and reproductive factors, prompting management actions such as nest protection and the establishment of a second population [74]. Similarly, analyses of more than 182 000 birds in global *ex situ* collections revealed substantial variation in sex ratios among institutions, highlighting how holding capacity, compatibility among individuals, reproductive management and exchanges between institutions can influence the demographic potential of managed avian populations [75]. Together, these examples support the interpretation that some *ex situ* parrot populations may be maintained through demographic supplementation and redistribution within the zoological network rather than through autonomous growth. This has direct implications for cooperative population management because continued coordination among institutions may be necessary to maintain population numbers when reproduction alone does not generate sufficient demographic replacement [73,75].

Our second hypothesis predicted that threatened species would perform better in captivity, with VU, EN and CR species showing higher reproductive output or population growth than less threatened species, potentially reflecting greater management and conservation attention. The results provided only partial support for this prediction. IUCN category was associated with reproductive output, but the magnitude and pattern of the differences did not indicate a consistent increase in reproductive performance with increasing extinction risk. At the mean year, predicted hatching rates ranged from 0.040 in LC species to 0.055 and 0.062 in EN and VU species, respectively. Thus, the estimated rates for EN and VU species were approximately 38 and 55% higher than those of LC species, respectively. Although these differences are biologically measurable, the absolute differences remained relatively small, and higher hatching rates did not consistently translate into positive population growth because mortality, acquisitions and other demographic processes also contributed to NetChange_pc. The lack of consistent differences for CR species further indicates that higher extinction risk was not associated with uniformly higher reproductive output.

The absence of a consistent relationship between threat status and population growth is particularly relevant for *ex situ* conservation planning. A threatened species may have a relatively productive captive population without necessarily having the demographic capacity to increase rapidly to a size suitable for reintroduction or reinforcement. This distinction matters for parrots because reproductive and life-history traits vary substantially among species. For example, an analysis of 260 captive parrot species found marked interspecific variation in lifespan and reproductive patterns, with more threatened species generally showing longer lifespans, later ages at last breeding and longer periods of active reproduction than species classified as LC or NT [20]. Thus, a higher threat category does not necessarily correspond to lower reproductive performance in captivity, and demographic potential cannot be inferred directly from IUCN status. Conversely, the management of captive parrot populations has historically involved species-specific breeding programmes, regional stud books and coordinated collection planning, reflecting the need to manage populations according to their demographic characteristics and conservation objectives rather than threat status alone [76]. The importance of management context is also illustrated by the international trade in Australian endemic parrots, where captive breeding outside Australia represented the major source of traded birds and species that were more abundant in captivity tended to occur in higher trade volumes, showing how availability within *ex situ* populations can differ substantially among species independently of their conservation status [77]. Therefore, IUCN category should not be used as the sole proxy for demographic performance in zoological collections. Instead, conservation planning should consider IUCN status alongside species-specific information on reproductive output, mortality, age structure, sex composition, genetic diversity, available space and the feasibility of coordinated population management.

Several threatened taxa, including the grey-breasted parakeet (CR), blue-headed macaw (EN), white-bellied parrot (*Pionites leucogaster*; EN) and golden parakeet (VU), exhibited positive NetChange_pc values associated with hatchings, whereas some less threatened species showed greater instability and reliance on acquisitions. These species-level patterns illustrate why statistical differences among IUCN categories should not be interpreted as direct measures of conservation success. The magnitude, persistence and demographic origin of population change are more informative for management than threat category alone. The case of Spix's macaw illustrates both the potential and the limitations of intensive management: although its fitted trajectory showed a positive but non-significant trend, its persistence remains dependent on coordinated genetic management and sustained institutional investment and support [78,79].

Our third hypothesis predicted that sex ratios closer to parity would be associated with greater population stability or growth. This prediction was partially supported. Sex ratio was nonlinearly associated with NetChange_pc, but the biological importance of this relationship appears limited at the population level. The model had a low adjusted *R*^2^ (0.013) and explained only 1.98% of the deviance, indicating that sex ratio accounted for only a small fraction of the observed variation in population change. Thus, although the relationship was statistically detectable, sex ratio alone had limited explanatory power for demographic performance.

The fitted relationship suggested higher NetChange_pc at balanced to moderately female-biased sex ratios, peaking around 0.45. However, broad CIs and sparse observations at extreme sex ratios warrant caution in interpreting this pattern as a management optimum. This association is consistent with demographic theory indicating that the consequences of sex-ratio variation depend on interactions among sex ratio, mating system, population size and demographic stochasticity, rather than on sex composition alone [80–83]. In birds, adult sex ratios also relate to mating systems and patterns of parental care, although these relationships vary among species, and the components of sex-specific reproductive roles can evolve independently [84]. Similarly, variation in sex composition can affect mate choice, sexual conflict, parental investment and social organization, making the demographic consequences of a given sex ratio dependent on species-specific reproductive and behavioural characteristics [85]. These findings provide a biological basis for the observed association between sex ratio and NetChange_pc, but they also indicate why captive parrot populations should not universally target a sex ratio of approximately 0.45. Rather, the observed nonlinear relationship suggests that substantial departures from balanced sex composition may constrain demographic performance in some populations, particularly where suitable mates are limited or where reproductive opportunities depend strongly on pair formation and social compatibility. Species-specific mating systems, pair-bonding behaviour, reproductive compatibility, age structure and social organization should therefore be considered before translating sex-ratio patterns into collection-management recommendations [83–85]. The model's low explanatory power further suggests that sex ratio represents only one component of a much broader set of demographic and management factors influencing population change.

Our fourth hypothesis predicted that international trade would decline with increasing extinction risk because of regulatory restrictions and conservation constraints. This prediction was not supported. Instead, the ordinal threat-level variable showed a positive association with trade volume. The estimated coefficient for threat level was 0.056, corresponding to approximately 1.06 times the predicted trade volume for each one-category increase in the ordinal threat scale, after accounting for the other variables. Thus, each additional increase in threat level was associated with only a modest change in predicted trade volume. Despite its statistical significance, this relationship should not be interpreted as strong or deterministic. Rather, it indicates a gradual increase in recorded trade with increasing threat level, potentially reflecting greater commercial or conservation interest in species of higher conservation concern.

By contrast, several other predictors showed substantially larger differences in predicted trade volume. Wild-caught specimens had an estimated trade volume approximately 2.2 times that of the reference source category, while commercial transactions were associated with approximately 2.8 times the volume of the reference purpose. Asia also showed approximately 3.5 times the predicted import volume relative to the African reference region. These results indicate that the association with threat level was comparatively modest, whereas source, purpose and geographical region showed much larger differences in recorded trade volume. Therefore, threat status alone cannot explain the biological and conservation implications of trade.

Wild-caught specimens were strongly associated with recorded trade and therefore remain particularly relevant to conservation. However, this finding should not be interpreted as evidence that zoological institutions themselves are responsible for wild capture. Rather, such individuals may enter zoological collections after confiscation or other enforcement actions associated with wildlife trafficking [78,86]. Their subsequent incorporation into *ex situ* populations can increase population size or potentially contribute genetic diversity, but unplanned acquisition may also introduce challenges related to health status, sanitary risks, unknown provenance and genetic management [87,88]. From a management perspective, distinguishing planned transfers from reactive incorporation of confiscated animals is therefore important when assessing whether increases in captive population size represent strategic conservation gains.

Regional patterns further highlight strong global asymmetries in parrot trade. Asia emerged as the primary importing region, while Africa exported proportionally more. The stronger association observed for Asia may reflect the emergence of western and Southeast Asian countries as important hubs in the international parrot trade, where demand for captive birds and companion animals has increased and established commercial networks connect Asian markets with major exporting regions such as Africa and South Africa [22,89,90]. Previous studies have similarly identified Asia as an increasingly important destination for internationally traded parrots and have linked import volumes to socio-economic characteristics of importing countries, including gross domestic product *per capita* [91,92]. By contrast, several African countries have historically functioned as important source regions for wild-caught parrots, particularly species such as the African grey parrot and other African parrot species [93–95]. South Africa has also become an important exporter of captive-bred parrots to Asian and Middle Eastern markets, supported by an established captive breeding industry [90]. These established source and destination networks may therefore contribute to the regional differences observed in our model. Trade dynamics are also likely to reflect interacting cultural, economic, regulatory and market factors, including demand for rare or aesthetically valued birds and species traits associated with their attractiveness to consumers [96,97]. However, because our analysis did not distinguish the specific purposes or market segments underlying these regional patterns, these mechanisms should be considered plausible explanations rather than direct inferences from our data.

Taken together, the four hypotheses indicate that the demographic and trade dynamics of captive Brazilian parrots are more complex than extinction risk or any single demographic variable alone would suggest. The first hypothesis received limited support because captive populations did not show consistent demographic growth and reproductive output declined over time. The second received partial support because some threatened categories showed higher predicted hatching rates than LC species, but the pattern was not consistent across threat categories, and the absolute differences were modest. The third identified a statistically detectable nonlinear association between sex ratio and population change, but its low explanatory power limits its biological interpretation. The fourth hypothesis was not supported because trade increased rather than decreased with increasing threat level, although the effect was modest relative to source, purpose and geographical predictors. These results collectively indicate that conservation value cannot be inferred from statistical significance or threat status alone; it also requires consideration of the magnitude, persistence, demographic origin and management context of population change.

Integrating ZIMS demographic data with CITES trade records and IUCN threat assessments therefore provides a broader perspective on *ex situ* conservation patterns. Some statistically significant effects were relatively small and explained little of the variation in demographic performance, whereas other effects, particularly those involving temporal reproductive decline and the source and purpose of traded specimens, were larger and potentially more consequential for management. Distinguishing these effect sizes matters because conservation decisions ultimately depend on whether observed differences are large enough, persistent and mechanistically plausible to alter population trajectories.

Despite the relevance of these findings, several limitations warrant acknowledgement. An important conceptual limitation is that the demographic indicators analysed here should not be interpreted as a comprehensive assessment of *ex situ* population viability. Population size, reproductive output, mortality, NetChange_pc and trade patterns provide useful indicators of demographic performance, but long-term viability also depends on factors that were not available or evaluated in this study, including genetic diversity, founder representation, age structure, reproductive potential, genetic management and coordinated population planning. A population may therefore show stable or positive demographic trends while remaining vulnerable to genetic erosion, skewed age or sex structure, limited reproductive potential or inadequate founder representation. Conversely, a population showing low or negative short-term demographic change may still have conservation value if it is genetically well managed, represents important founder lineages or fulfils a specific role within a coordinated *ex situ* programme. Our results should therefore be interpreted as an assessment of demographic performance and associated trade patterns rather than as a complete evaluation of long-term population viability.

ZIMS data also vary in institutional coverage and record completeness, with holdings concentrated in zoological institutions in Europe, North America and Asia; consequently, collections and facilities in species-rich regions, such as South America and Africa, may be under-represented [98,99]. Taxonomic inconsistencies and differences in taxonomic resolution across datasets may also affect record matching and, consequently, bias demographic and wildlife-trade estimates [99,100]. Importantly, ZIMS aggregates records from institutions operating under different husbandry, breeding-management and population-management conditions. Differences in institutional participation and reporting completeness may therefore contribute to unexplained variation in demographic performance. Although the models accounted for repeated observations and between-species heterogeneity, the available data did not allow direct evaluation of institution-level management practices or species-specific biological traits. Reproductive output is also not necessarily maximized in captivity; for some abundant species, reproduction may be intentionally limited because of space constraints, population-management priorities or low institutional demand, potentially influencing observed temporal population trends.

The global GAMs of *per capita* population change did not meet the predefined diagnostic criteria and were therefore reported for descriptive purposes only, with formal inference based on genus-level models that met the diagnostic criteria. The GAMM relating sex ratio to *per capita* population change had low explanatory power (adjusted *R*^2^ = 0.013; deviance explained = 1.98%), reinforcing that the statistically significant association should not be interpreted as a major determinant of population dynamics. Future research should integrate species-specific traits with management data, including age structure, reproductive maturity, pairing success, enclosure capacity, social composition, genetic management and breeding-programme recommendations. These data would help distinguish statistically detectable patterns from demographic changes large and persistent enough to affect *ex situ* conservation.

Future studies should assess whether management conditions generate sustained demographic surpluses rather than relying on population counts alone. This would allow researchers to determine whether apparently stable populations are genuinely self-sustaining or instead depend on continued transfers and acquisitions. Integrating demographic records with institution-level management data would be particularly valuable for identifying which husbandry and breeding strategies are associated with sustained reproductive performance and whether these effects differ among species with contrasting life histories. Such integration could also strengthen coordinated population planning by allowing regional breeding programmes to identify demographic bottlenecks, prioritize breeding opportunities, coordinate transfers and align institutional capacity with species-specific conservation objectives [101]. Combining demographic performance with information on genetic diversity, age and sex structure, reproductive compatibility, available space and population-management recommendations would provide a stronger basis for evaluating each institution's contribution to coordinated *ex situ* conservation programmes.

These findings also have practical implications for coordinated regional and international population-management programmes. For zoo professionals and conservation practitioners, the results suggest that population planning should extend beyond maintaining adequate numbers at individual institutions and instead consider each species’ demographic performance and management needs across the wider *ex situ* network. Species showing low reproductive output or persistent dependence on population supplementation may require coordinated breeding plans that identify compatible breeding pairs, optimize the distribution of reproductive individuals among institutions and anticipate future demographic needs. In this context, transfers can be used strategically to balance sex composition, age structure, breeding opportunities and available space across participating institutions, rather than simply to compensate for local population declines. However, because transfers do not necessarily add individuals or genetic diversity to the overall *ex situ* population, their conservation value should be evaluated in relation to coordinated population planning and genetic management.

At a regional scale, demographic information from multiple institutions could help identify populations productive enough to serve as sources for other facilities, while reducing unnecessary duplication of breeding efforts. Such coordination may be particularly relevant for threatened species, for which the relatively higher hatching rates observed in some IUCN categories did not consistently translate into positive population growth. Population-management programmes could therefore use demographic indicators such as reproductive output, mortality, NetChange_pc, sex composition and dependence on transfers to identify management priorities and evaluate whether captive populations are progressing towards self-sustaining demographic trajectories. Integrating these indicators with studbook information, genetic management, space availability and species-specific life-history characteristics would provide a more comprehensive basis for regional breeding programmes and wider *ex situ* conservation strategies.

## 6. Conclusion

This study provides quantitative evidence that *ex situ* populations of Brazilian parrot species, while broadly maintained across zoological institutions over two decades, were not consistently self-sustaining through reproduction alone. Their demographic performance should, however, not be equated with long-term population viability, which also depends on genetic diversity, founder representation, age structure, reproductive potential and coordinated population management. Hatching rates declined over time, while many species showed stable or negative *per capita* population change and depended substantially on acquisitions or transfers to maintain population size at the institutional level. However, these movements do not necessarily represent additions to the overall *ex situ* population or increases in genetic diversity, as many acquisitions may involve individuals already maintained elsewhere within the zoological network.

Threat status alone was not a strong predictor of captive demographic performance. Although some threatened categories had higher predicted hatching rates than LC species, the absolute differences were relatively modest and did not translate consistently into positive population growth. Similarly, the statistically significant association between sex ratio and NetChange_pc explained only a small proportion of demographic variation, indicating that sex composition may contribute to population performance but is unlikely to act as a major determinant in isolation. By contrast, the magnitude of some trade-related associations, particularly those involving specimen origin, transaction purpose and geographical region, indicates greater practical relevance for understanding international movements of psittacine species.

Together, these findings emphasize that demographic performance is only one component of *ex situ* conservation. The conservation value of managed populations should be considered in relation to the magnitude, direction, persistence and demographic origin of population change, as well as genetic diversity, founder representation, age and sex structure, reproductive potential, animal welfare and the intended conservation function of each population. Strengthening the *ex situ* conservation of parrots will therefore require coordinated population planning across institutions and regions, combining demographic, genetic, welfare and management information. In particular, demographic information can help identify populations needing reproductive support, opportunities for strategic transfers and species for which coordinated breeding efforts may be relevant, while distinguishing local increases from transfers from demographic growth through reproduction. Future studies combining long-term demographic records with species-specific biological traits, genetic information, and detailed husbandry and management data will be important for determining which captive populations can contribute to recovery, reinforcement or reintroduction programmes.

## Supplementary Material



## Data Availability

All data generated or analysed in this study are available online via the Mendeley Data repository [47]. Supplementary material is available online [102].

## References

[1] Berkunsky I *et al.* 2017 Current threats faced by neotropical parrot populations. Biol. Conserv. 214, 278–287. (doi:10.1016/j.biocon.2017.08.016)

[2] Vergara-Tabares DL , Cordier JM, Landi MA, Olah G, Nori J. 2020 Global trends of habitat destruction and consequences for parrot conservation. Glob. Chang. Biol. 26, 4251–4262. (doi:10.1111/gcb.15135) 32338813

[3] Destro GFG , de Fernandes V, de Andrade AFA, De Marco P, Terribile LC. 2019 Back home? Uncertainties for returning seized animals to the source-areas under climate change. Glob. Chang. Biol. 25, 3242–3253. (doi:10.1111/gcb.14760) 31306533

[4] Owens IPF , Bennett PM. 2000 Ecological basis of extinction risk in birds: habitat loss versus human persecution and introduced predators. Proc. Natl Acad. Sci. USA 97, 12144–12148. (doi:10.1073/pnas.200223397) PMC17308 11005835

[5] Pires SF , Olah G, Nandika D, Agustina D, Heinsohn R. 2021 What drives the illegal parrot trade? Applying a criminological model to market and seizure data in Indonesia. Biol. Conserv. 257, 109098. (doi:10.1016/j.biocon.2021.109098)

[6] Sánchez-Mercado A , Ferrer-Paris JR, Rodríguez JP, L. Tella J. 2021 A literature synthesis of actions to tackle illegal parrot trade. Diversity (Basel) 13, 191. (doi:10.3390/d13050191)

[7] Azeredo LMM , Alves RRN. 2024 Understanding the drivers of the live bird trade in Brazil. Ethnobiol. Conserv. 13, 1–25. (doi:10.15451/ec2024-01-13.05-1-19)

[8] Vanstreels RET , Teixeira RHF, Camargo LC, Nunes ALV, Matushima ER. 2010 Impacts of animal traffic on the Brazilian Amazon parrots (*Amazona* species) collection of the Quinzinho de Barros Municipal Zoological Park, Brazil, 1986-2007. Zoo Biol. 29, 600–614. (doi:10.1002/zoo.20300) 20069544

[9] Vilarta MR , Wittkoff W, Lobato C, Oliveira RdA, Pereira NGP, Silveira LF. 2021 Reintroduction of the golden conure (*Guaruba guarouba*) in northern Brazil: establishing a population in a protected area. Diversity (Basel) 13, 198. (doi:10.3390/d13050198)

[10] McClure CJW *et al.* 2023 Conserving the evolutionary history of birds. Conserv. Biol. 37, e14141. (doi:10.1111/cobi.14141) 37424371

[11] Bolam FC *et al.* 2021 How many bird and mammal extinctions has recent conservation action prevented? Conserv. Lett. 14, e12762. (doi:10.1111/conl.12762)

[12] Powell DM *et al.* 2025 Advancing animal welfare: the world zoo and aquarium animal welfare strategy. Barcelona, Spain: World Association of Zoos and Aquariums.

[13] Schwartz KR , Parsons ECM, Rockwood L, Wood TC. 2017 Integrating *in-situ* and *ex*-*situ* data management processes for biodiversity conservation. Front. Ecol. Evol. 5, 120. (doi:10.3389/fevo.2017.00120)

[14] Species360 Zoological Information Management System (ZIMS) . 2025 Species360 Zoological Information Management System (ZIMS). See zims.Species360.org.

[15] CITES . 2026 CITES - Convention on International Trade in Endangered species of wild Fauna and Flora. See https://cites.org/eng. 10.1159/000459796 712806

[16] Harfoot MBJ *et al.* 2021 Using the IUCN Red List to map threats to terrestrial vertebrates at global scale. Nat. Ecol. Evol. 5, 1510–1519. (doi:10.1038/s41559-021-01542-9) PMC8560638 34462602

[17] IUCN . 2026 *The IUCN Red List of Threatened Species*, Version 2025-2. See https://www.iucnredlist.org.

[18] Green J , Schmidt-Burbach J, Elwin A. 2022 Commercial trade of wild animals: examining the use of the IUCN Red List and CITES appendices as the basis for corporate trade policies. Front. Conserv. Sci. 3, 902074. (doi:10.3389/fcosc.2022.902074)

[19] Andersson AA , Tilley HB, Lau W, Dudgeon D, Bonebrake TC, Dingle C. 2021 CITES and beyond: illuminating 20 years of global, legal wildlife trade. Glob. Ecol. Conserv. 26, e01455. (doi:10.1016/j.gecco.2021.e01455)

[20] Young AM , Hobson EA, Lackey LB, Wright TF. 2012 Survival on the ark: life-history trends in captive parrots. Anim. Conserv. 15, 28–43. (doi:10.1111/j.1469-1795.2011.00477.x) PMC3289156 22389582

[21] Frynta D , Lišková S, Bültmann S, Burda H. 2010 Being attractive brings advantages: the case of parrot species in captivity. PLoS ONE 5, 1–9. (doi:10.1371/journal.pone.0012568) PMC2935353 20830206

[22] Chan DTC , Poon ESK, Wong ATC, Sin SYW. 2021 Global trade in parrots – influential factors of trade and implications for conservation. Glob. Ecol. Conserv. 30, e01784. (doi:10.1016/j.gecco.2021.e01784)

[23] Campos CI , Martinez MA, Acosta D, Diaz-Luque JA, Berkunsky I, Lamberski NL, Cruz-Nieto J, Russello MA, Wright TF. 2021 Genetic diversity and population structure of two endangered neotropical parrots inform *in situ* and *ex situ* conservation strategies. Diversity (Basel) 13, 386. (doi:10.3390/d13080386)

[24] Russello MA , Amato G. 2004 *Ex situ* population management in the absence of pedigree information. Mol. Ecol. 13, 2829–2840. (doi:10.1111/j.1365-294X.2004.02266.x) 15315693

[25] Gelman A . 2008 Scaling regression inputs by dividing by two standard deviations. Stat. Med. 27, 2865–2873. (doi:10.1002/sim.3107) 17960576

[26] Schielzeth H . 2010 Simple means to improve the interpretability of regression coefficients. Methods Ecol. Evol. 1, 103–113. (doi:10.1111/j.2041-210X.2010.00012.x)

[27] Zuur AF , Ieno EN, Walker NJ, Saveliev AA, Smith GM. 2009 Mixed effects models and extensions in ecology with R. New York, NY: Springer.

[28] Munoz A , Carey V, Schouten JP, Segal M, Rosner B. 1992 A parametric family of correlation structures for the analysis of longitudinal data. Biometrics 48, 733–742. (doi:10.2307/2532340) 1420837

[29] Simpson SL , Edwards LJ, Muller KE, Sen PK, Styner MA. 2010 A linear exponent AR(1) family of correlation structures. Stat. Med. 29, 1825–1838. (doi:10.1002/sim.3928) PMC4020183 20658550

[30] Zavrakidis I , Jóźwiak K, Hauptmann M. 2020 Statistical analysis of longitudinal data on tumour growth in mice experiments. Sci. Rep. 10, 9143. (doi:10.1038/s41598-020-65767-7) PMC7272435 32499558

[31] Liang K-Y , Zeger SL. 1986 Longitudinal data analysis using generalized linear models. Biometrika 73, 13–22. (doi:10.1093/biomet/73.1.13)

[32] McCullagh P , Nelder JA. 2019 Generalized linear models, 2nd edn. New York, NY: Routledge. (doi:10.1201/9780203753736)

[33] Cameron AC , Trivedi PK. 2013 Regression analysis of count data, 2nd edn. Cambridge, UK: Cambridge University Press. (doi:10.1017/CBO9781139013567)

[34] Hartig F . 2022 DHARMa: residual diagnostics for hierarchical (multi-level/mixed) regression models. See https://CRAN.R-project.org/package=DHARMa.

[35] Wood S . 2023 *mgcv: mixed GAM computation vehicle with automatic smoothness estimation*. See https://CRAN.R-project.org/package=mgcv.

[36] R Core Team . 2025 R: a language and environment for statistical computing.Vienna, Austria: R Foundation for Statistical Computing.

[37] Brooks ME , Kristensen K, Benthem KJ, van Magnusson A, Berg CW, Nielsen A, Skaug HJ, Mächler M, Bolker BM. 2017 glmmTMB balances speed and flexibility among packages for zero-inflated generalized linear mixed modeling. R J. 9, 378. (doi:10.32614/RJ-2017-066)

[38] Lüdecke D , Ben-Shachar M, Patil I, Waggoner P, Makowski D. 2021 performance: an R package for assessment, comparison and testing of statistical models. J. Open Source Softw. 6, 3139. (doi:10.21105/joss.03139)

[39] Lenth RV *et al.* 2024 Emmeans: estimated marginal means, aka least-squares means. See https://CRAN.R-project.org/package=emmeans.

[40] Wickham H *et al.* 2019 Welcome to the Tidyverse. J. Open Source Softw. 4, 1686. (doi:10.21105/joss.01686)

[41] Wickham H , François R, Henry L, Müller K. 2022 Package dplyr. R package version 2.2-0. See https://cran.r-project.org/web/packages/dplyr/index.html.

[42] Wickham H . 2016 ggplot2: elegant graphics for data analysis, 2nd edn. Cham, Switzerland: Springer International Publishing. (doi:10.1007/978-3-319-24277-4)

[43] Simpson GL . 2024 gratia: an R package for exploring generalized additive models. J. Open Source Softw. 9, 6962. (doi:10.21105/joss.06962)

[44] Arel-Bundock V , Enevoldsen N, Yetman C. 2018 countrycode: an R package to convert country names and country codes. J. Open Source Softw. 3, 848. (doi:10.21105/joss.00848)

[45] Firke S . 2024 Janitor: simple tools for examining and cleaning dirty data. See https://CRAN.R-project.org/package=janitor.

[46] Breheny P , Burchett W. 2017 Visualization of regression models using visreg. R J. 9, 56–71. (doi:10.32614/RJ-2017-046)

[47] Baltazar I , Schork I, Figueiredo Passos L, Ramos G, Pires MRS, de Azevedo CS. 2026 *Ex-situ* reproduction and population viability of Brazilian psittacines: integrating data from zoos, international trade, and conservation status. Mendeley data v1. (doi:10.17632/t57y7tt4f8.1)

[48] Earnhardt J , Vélez‐Valentín J, Valentin R, Long S, Lynch C, Schowe K. 2014 The Puerto Rican parrot reintroduction program: sustainable management of the aviary population. Zoo Biol. 33, 89–98. (doi:10.1002/zoo.21109) 24395187

[49] Che‐Castaldo J *et al.* 2019 Patterns in the long-term viability of North American zoo populations. Zoo Biol. 38, 78–94. (doi:10.1002/zoo.21471) 30609112

[50] Che-Castaldo J , Gray SM, Rodriguez-Clark KM, Schad Eebes K, Faust LJ. 2021 Expected demographic and genetic declines not found in most zoo and aquarium populations. Front. Ecol. Environ. 19, 435–442. (doi:10.1002/fee.2362)

[51] Leimgruber P , Senior B, Uga, Aung M, Songer MA, Mueller T, Wemmer C, Ballou JD. 2008 Modeling population viability of captive elephants in Myanmar (Burma): implications for wild populations. Anim. Conserv. 11, 198–205. (doi:10.1111/j.1469-1795.2008.00172.x)

[52] Zeoli LF , Sayler RD, Wielgus R. 2008 Population viability analysis for captive breeding and reintroduction of the endangered Columbia basin pygmy rabbit. Anim. Conserv. 11, 504–512. (doi:10.1111/j.1469-1795.2008.00208.x)

[53] Suter IC , Maurer GP, Baxter G. 2014 Population viability of captive Asian elephants in the Lao PDR. Endanger. Species Res. 24, 1–7. (doi:10.3354/esr00578)

[54] Scott SE , Cain B, de Kort SR, Johnson B, Khayale C, Versteege L, Bettridge CM. 2023 Group composition impacts reproductive output and population viability in captive white rhinoceros. Anim. Conserv. 26, 290–302. (doi:10.1111/acv.12833)

[55] Renton K , Salinas-Melgoza A, De Labra-Hernández MÁ, de la Parra-Martínez SM. 2015 Resource requirements of parrots: nest site selectivity and dietary plasticity of Psittaciformes. J. Ornithol. 156, 73–90. (doi:10.1007/s10336-015-1255-9)

[56] Robert A . 2009 Captive breeding genetics and reintroduction success. Biol. Conserv. 142, 2915–2922. (doi:10.1016/j.biocon.2009.07.016)

[57] Conde DA , Colchero F, Gusset M, Pearce-Kelly P, Byers O, Flesness N, Browne RK, Jones OR. 2013 Zoos through the lens of the IUCN red list: a global metapopulation approach to support conservation breeding programs. PLoS ONE 8, e80311. (doi:10.1371/journal.pone.0080311) PMC3859473 24348999

[58] Bussolini LT , Crates R, Magrath MJL, Stojanovic D. 2023 Identifying factors affecting captive breeding success in a critically endangered species. Emu – Austral. Ornithol. 123, 161–169. (doi:10.1080/01584197.2023.2194541)

[59] Bradshaw GA , Engebretson M. 2013 Parrot breeding and keeping: the impact of capture and captivity, 1st edn. Miami, FL: Animals and Society Institute. See www.animalsandsociety.org.

[60] Sick H . 1997 Ornitologia brasileira. Rio de Janeiro, Brazil: Nova Fronteira.

[61] Sigrist T . 2013 Avifauna brasileira. Vinhedo, Brazil: Avis Brasilis.

[62] Forshaw JM . 2010 Parrots of the world. Princeton, NJ: Princeton University Press.

[63] Faust LJ , Long ST, Perišin K, Simonis JL. 2019 Uncovering challenges to sustainability of AZA animal programs by evaluating the outcomes of breeding and transfer recommendations with PMCTrack. Zoo Biol. 38, 24–35. (doi:10.1002/zoo.21470) 30614074

[64] IUCN/SSC . 2025 IUCN SSC WPaSG guidelines. See https://www.iucn-wildparrot-specialist-group.org/home/iucn-ssc-wpasg-guidelines#h.lrzj5j9g2f3z.

[65] Gray SM , Faust LJ, Kuykendall NA, Bladow RA, Schad Eebes K, Che-Castaldo JP. 2022 Reasons for unfulfilled breeding and transfer recommendations in zoos and aquariums. Zoo Biol. 41, 143–156. (doi:10.1002/zoo.21664) PMC9299711 34870877

[66] Lacy RC . 2013 Achieving true sustainability of zoo populations. Zoo Biol. 32, 19–26. (doi:10.1002/zoo.21029) 22753040

[67] Sheppard C . 1995 Propagation of endangered birds in US institutions: how much space is there? Zoo Biol. 14, 197–210. (doi:10.1002/zoo.1430140302)

[68] Martin‐Wintle MS , Wintle NJP, Díez‐León M, Swaisgood RR, Asa CS. 2019 Improving the sustainability of *ex situ* populations with mate choice. Zoo Biol. 38, 119–132. (doi:10.1002/zoo.21450) 30474268

[69] Greggor AL , Vicino GA, Swaisgood RR, Fidgett A, Brenner D, Kinney ME, Farabaugh S, Masuda B, Lamberski N. 2018 Animal welfare in conservation breeding: applications and challenges. Front. Vet. Sci. 5, 323. (doi:10.3389/fvets.2018.00323) PMC6315189 30631770

[70] Marshall AR , Deere NJ, Little HA, Snipp R, Goulder J, Mayer-Clarke S. 2016 Husbandry and enclosure influences on penguin behavior and conservation breeding. Zoo Biol. 35, 385–397. (doi:10.1002/zoo.21313) 27486862

[71] Flanagan AM , Masuda B, Grabar K, Barrett LP, Swaisgood RR. 2024 An enclosure quality ranking framework for terrestrial animals in captivity. Appl. Anim. Behav. Sci. 278, 106378. (doi:10.1016/j.applanim.2024.106378)

[72] Carlstead K , Shepherdson D. 1994 Effects of environmental enrichment on reproduction. Zoo Biol. 13, 447–458. (doi:10.1002/zoo.1430130507)

[73] Powell DM , Dorsey CL, Faust LJ. 2019 Advancing the science behind animal program sustainability: an overview of the special issue. Zoo Biol. 38, 5–11. (doi:10.1002/zoo.21474) 30629303

[74] Beissinger SR , Wunderle JM, Meyers JM, Sæther B-E, Engen S. 2008 Anatomy of a bottleneck: diagnosing factors limiting population growth in the Puerto Rican parrot. Ecol. Monogr. 78, 185–203. (doi:10.1890/07-0018.1)

[75] Hall CA , Conroy GC, Potvin DA. 2025 *Ex-situ* avian sex skews: determinants and implications for conservation. PeerJ 13, e19312. (doi:10.7717/peerj.19312) PMC12011015 40260192

[76] Wilkinson R . 2000 An overview of captive-management programmes and regional collection planning for parrots. Int. Zoo Yearbook 37, 36–58. (doi:10.1111/j.1748-1090.2000.tb00705.x)

[77] Vall-llosera M , Cassey P. 2017 ‘Do you come from a land down under?’ Characteristics of the international trade in Australian endemic parrots. Biol. Conserv. 207, 38–46. (doi:10.1016/j.biocon.2017.01.015)

[78] Lugarini C , Vercillo U, Purchase C, Watson R, Schischakin N. 2021 A Conservação da Ararinha-azul, *Cyanopsitta spixii* (Wagler, 1832): Desafios e Conquistas. Biodiversidade Brasileira - BioBrasil 11, 1–16. (doi:10.37002/biobrasil.v11i3.1746)

[79] Vercillo U *et al.* 2023 Spix's macaw *Cyanopsitta spixii* (Wagler, 1832) population viability analysis. Bird Conserv. Int. 33, e67. (doi:10.1017/S0959270923000217)

[80] Snyder N , McGowan P, Gilardi J, Grajal A. 2000 Parrots. Status survey and conservation action plan 2000–2004. Gland, Switzerland: IUCN.

[81] Snyder NFR , Derrickson SR, Beissinger SR, Wiley JW, Smith TB, Toone WD, Miller B. 1996 Limitations of captive breeding in endangered species recovery. Conserv. Biol. 10, 338–348. (doi:10.1046/j.1523-1739.1996.10020338.x)

[82] Payevsky VA . 2021 Sex ratio and sex-specific survival in avian populations: a review. Biol. Bull. Rev. 11, 317–327. (doi:10.1134/S2079086421030099)

[83] Lee AM , Sæther BE, Engen S. 2011 Demographic stochasticity, Allee effects, and extinction: the influence of mating system and sex ratio. Am. Nat. 177, 301–313. (doi:10.1086/658344) 21460539

[84] Gonzalez‐Voyer A , Thomas GH, Liker A, Krüger O, Komdeur J, Székely T. 2022 Sex roles in birds: phylogenetic analyses of the influence of climate, life histories and social environment. Ecol. Lett. 25, 647–660. (doi:10.1111/ele.13938) 35199926

[85] Kappeler PM *et al.* 2023 Sex roles and sex ratios in animals. Biol. Rev. 98, 462–480. (doi:10.1111/brv.12915) 36307924

[86] Sampaio MB , Schiel N, Souto A. 2020 From exploitation to conservation: a historical analysis of zoos and their functions in human societies. Ethnobiol. Conserv. 9, 1–32. (doi:10.15451/EC2020-01-9.02-1-32)

[87] Woodford MH . 2000 Quarantine and health screening protocols for wildlife prior to translocation and release into the wild. Other Public. Zoonot. Wildl. Dis. 32, 1–88.

[88] Olah G , Smith BT, Joseph L, Banks SC, Heinsohn R. 2021 Advancing genetic methods in the study of parrot biology and conservation. Diversity (Basel) 13, 521. (doi:10.3390/d13110521)

[89] Bush ER , Baker SE, Macdonald DW. 2014 Global trade in exotic pets 2006-2012. Conserv. Biol. 28, 663–676. (doi:10.1111/cobi.12240) 24661260

[90] Shivambu N , Shivambu TC, Nelufule T, Moshobane MC, Seoraj-Pillai N, Nangammbi TC. 2024 South Africa as a donor of its native and exotic species to the international pet trade. Sustainability (Switzerland) 16, 8828. (doi:10.3390/su16208828)

[91] Yin RY , Ye YC, Newman C, Buesching CD, Macdonald DW, Luo Y, Zhou ZM. 2020 China's online parrot trade: generation length and body mass determine sales volume via price. Glob. Ecol. Conserv. 23, e01047. (doi:10.1016/j.gecco.2020.e01047) PMC7144616 32292803

[92] Poole CM , Shepherd CR. 2017 Shades of grey: the legal trade in CITES-listed birds in Singapore, notably the globally threatened African grey parrot *Psittacus erithacus*. ORYX 51, 411–417. (doi:10.1017/S0030605314000234)

[93] Martin RO . 2017 Grey areas: temporal and geographical dynamics of international trade of grey and Timneh parrots (*Psittacus erithacus* and *P. timneh*) under CITES. Emu 118, 113–125. (doi:10.1080/01584197.2017.1369854)

[94] Martin RO , Senni C, D'Cruze NC. 2018 Trade in wild-sourced African grey parrots: insights via social media. Glob. Ecol. Conserv. 15, 00429. (doi:10.1016/j.gecco.2018.e00429)

[95] Martin RO . 2018 The wild bird trade and African parrots: past, present and future challenges. Ostrich 89, 139–143. (doi:10.2989/00306525.2017.1397787)

[96] Hausmann A , Cortés-Capano G, Fraser I, Di Minin E. 2023 Assessing preferences and motivations for owning exotic pets: care matters. Biol. Conserv. 281, 110007. (doi:10.1016/j.biocon.2023.110007)

[97] Haukka A , Jürgens J, Staerk J, Lehikoinen A, Bruslund S, Santangeli A. 2026 Aesthetic values predict bird trade, but the association varies across product types and trade regions. Biol. Conserv. 313, 111572. (doi:10.1016/j.biocon.2025.111572)

[98] Wahle A , Graßmann C, Zell E, Leiss L, Marcordes B, Pagel TB, Ziegler T. 2025 Non-passerine birds in zoos: a global approach on distribution patterns of threatened birds in zoological institutions. Integr. Conserv. 4, 600–615. (doi:10.1002/inc3.70043)

[99] Ahmed K , Tapley B, Michaels CJ. 2024 Global and regional patterns in distribution and threat status of zoo collections of turtles and tortoises. Herpetol. J. 34, 1–10. (doi:10.33256/34.1.110)

[100] Grenié M , Berti E, Carvajal‐Quintero J, Dädlow GML, Sagouis A, Winter M. 2023 Harmonizing taxon names in biodiversity data: a review of tools, databases and best practices. Methods Ecol. Evol. 14, 12–25. (doi:10.1111/2041-210X.13802)

[101] World Association of Zoos and Aquariums . 2015 The world zoo and aquarium conservation strategy committing to Conservtion. Gland, Switzerland: WAZA Executive Office.

[102] Baltazar I , Schork I, Figueiredo Passos L, Ramos G, Pires MRS, de Azevedo CS. 2026 Supplementary material from: *Ex-situ* reproduction and population viability of Brazilian psittacines: integrating data from zoos, international trade, and conservation status. Figshare. (doi:10.6084/m9.figshare.c.8784781)

