# Supplementary material for: Ex situ population dynamics of Brazilian parrots based on zoo, trade and conservation data
Source: R Soc Open Sci. 2026 Nov 11;13(11):261135. doi: 10.1098/rsos.261135 (PMC13750944; doi:10.1098/rsos.261135)
Supplement: Supplementary file 1 [file rsos.261135.zip › rsos.261135/rsos261135_si_001.docx]

**Supplementary Material 1.** Brazilian Psittacidae species assessed in the present study, according to different threat categories (IUCN, 2025): LC = Least Concern; NT = Near Threatened; VU = Vulnerable; EN = Endangered; CR = Critically Endangered; and EW = Extinct in the Wild. Appendices I and II refer to the level of protection established by CITES (2024): Appendix I includes species threatened with extinction, for which trade is permitted only under exceptional circumstances, whereas Appendix II encompasses species not necessarily threatened with extinction but whose trade must be regulated to prevent utilisation incompatible with their survival. The abbreviation N/A (not evaluated) indicates species not listed in the CITES appendices.

| **Common name** | **Scientific name** | **Threat level (IUCN)** | **CITES Appendix** |
| --- | --- | --- | --- |
| Yellow-faced parrot | *Alipiopsitta xanthops* (Spix, 1824) | NT | 2 |
| Blue-fronted Amazon parrot | *Amazona aestiva* (Linnaeus, 1758) | NT | 2 |
| Orange-winged Amazon parrot | *Amazona amazonica* (Linnaeus, 1766) | LC | 2 |
| Red-lored Amazon parrot | *Amazona autumnalis* (Linnaeus, 1758) | LC | 2 |
| Red-tailed Amazon parrot | *Amazona brasiliensis* (Linnaeus, 1758) | NT | 2 |
| Blue-cheeked Amazon parrot | *Amazona dufresniana* (Shaw, 1812) | NT | 2 |
| Mealy Amazon parrot | *Amazona farinosa* (Boddaert, 1783) | LC | 2 |
| Festive Amazon parrot | *Amazona festiva* (Linnaeus, 1758) | LC | 2 |
| Yellow-crowned Amazon parrot | *Amazona ochrocephala* (Gmelin, 1788) | LC | 2 |
| Red-spectacled Amazon parrot | *Amazona pretrei* (Temminck, 1830) | VU | 1 |
| Red-browd Amazon parrot | *Amazona rhodocorytha* (Salvadori, 1890) | VU | 1 |
| Vinaceous-breated Amazon parrot | *Amazona vinacea* (Kuhl, 1820) | EN | 1 |
| Hyacinth macaw | *Anodorhynchus hyacinthinus* (Latham, 1790) | VU | 1 |
| Lear’s macaw | *Anodorhynchus leari* Bonaparte, 1856 | EN | 1 |
| Blue-and-yellow macaw | *Ara ararauna* (Linnaeus, 1758) | LC | 2 |
| Red-and-green macaw | *Ara chloropterus*  Gray, 1859 | LC | 2 |
| Scarlet macaw | *Ara macao* (Linnaeus, 1758) | LC | 1 |
| Chestnut-fronted macaw | *Ara severus* (Linnaeus, 1758 | LC | 2 |
| Golden-capped parakeet | *Aratinga auricapillus* (Kuhl, 1820) | LC | 2 |
| Jandaya parakeet | *Aratinga jandaya (Gmelin, 1788)* | LC | 2 |
| Nanday parakeet | *Aratinga nenday (Vieillot, 1823)* | LC | N/A |
| Sun conure | *Aratinga solstitialis (Linnaeus, 1766)* | EN | 2 |
| Dusk-headed parakeet | *Aratinga weddellii (Deville, 1851)* | LC | 2 |
| Yellow-chefroned parakeet | *Brotogeris chiriri (Vieillot, 1818)* | LC | 2 |
| Cobalt-winged parakeet | *Brotogeris cyanoptera (Pelzeln, 1870)* | LC | 2 |
| Tui-parakeet | *Brotogeris sanctithomae (Statius Muller, 1776)* | LC | 2 |
| Plain parakeet | *Brotogeris tirica (Gmelin, 1788)* | LC | 2 |
| White-winged parakeet | *Brotogeris versicolurus (Statius Muller, 1776)* | LC | 2 |
| Spix’s macaw | *Cyanopsitta spixii (Wagler, 1832)* | EW | 1 |
| Red-fan parrot | *Deroptyus accipitrinus (Linnaeus, 1758)* | LC | 2 |
| Red-shouldered macaw | *Diopsittaca nobilis (Linnaeus, 1758)* | LC | 2 |
| Peach-fronted parakeet | *Eupsittula aurea (Gmelin, 1788)* | LC | 2 |
| Cactus parakeet | *Eupsittula cactorum (Kuhl, 1820)* | LC | 2 |
| Brown-throated parakeet | *Eupsittula pertinax (Linnaeus, 1758)* | LC | 2 |
| Green-rumped parrotlet | *Forpus passerinus (Linnaeus, 1758)* | LC | 2 |
| Short-tailed parrot | *Graydidascalus brachyurus (Kuhl, 1820)* | LC | 2 |
| Golden parakeet | *Guaruba guarouba (Gmelin, 1788)* | VU | 1 |
| Monk parakeet | *Myiopsitta monachus (Boddaert, 1783)* | LC | 2 |
| Red-bellied macaw | *Orthopsittaca manilatus (Boddaert, 1783)* | LC | 2 |
| White-bellied parrot | *Pionites leucogaster (Kuhl, 1820)* | EN | 2 |
| Black-headed parrot | *Pionites melanocephalus (Linnaeus, 1758)* | LC | 2 |
| Pileated parrot | *Pionopsitta pileata (Scopoli, 1769)* | LC | 1 |
| Dusk parrot | *Pionus fuscus (Statius Muller, 1776)* | LC | 2 |
| Scaly-headed parrot | *Pionus maximiliani (Kuhl, 1820)* | LC | 2 |
| Blue-headed parrot | *Pionus menstruus (Linnaeus, 1766)* | LC | 2 |
| Golden-collared macaw | *Primolius auricollis (Cassin, 1853)* | LC | 2 |
| Blue-headed macaw | *Primolius couloni (Sclater, 1876)* | VU | 1 |
| Blue-winged macaw | *Primolius maracana (Vieillot, 1816)* | NT | 1 |
| Blue-crowned parakeet | *Psittacara acuticaudatus (Vieillot, 1818)* | LC | 2 |
| White-eyed parakeet | *Psittacara leucophthalmus (Statius Muller, 1776)* | LC | 2 |
| Pearly parakeet | *Pyrrhura coerulescens Neumann, 1927* | VU | 2 |
| Ochre-marked parakeet | *Pyrrhura cruentata (Wied, 1820)* | VU | 1 |
| Fiery-shouldered parakeet | *Pyrrhura egregia (Sclater, 1881)* | LC | 2 |
| Maroon-bellied parakeet | *Pyrrhura frontalis (Vieillot, 1817)* | LC | 2 |
| Grey-breated parakeet | *Pyrrhura griseipectus Salvadori, 1900* | CR | 2 |
| White-eared parakeet | *Pyrrhura leucotis (Kuhl, 1820)* | NT | 2 |
| Maroon-tailed parakeet | *Pyrrhura melanura (Spix, 1824)* | LC | 2 |
| Green-cheeked parakeet | *Pyrrhura molinae (Massena & Souancé, 1854)* | LC | 2 |
| Crimson-bellied parakeet | *Pyrrhura perlata (Spix, 1824)* | VU | 2 |
| Painted parakeet | *Pyrrhura picta (Statius Muller, 1776)* | LC | 2 |
| Rose-fronted parakeet | *Pyrrhura roseifrons (Gray, 1859)* | LC | 2 |
| Black-capped parakeet | *Pyrrhura rupicola (Tschudi, 1844)* | NT | 2 |
| Blue-bellied parrot | *Triclaria malachitacea (Spix, 1824)* | NT | 2 |
