# Supplementary material for: Ex situ population dynamics of Brazilian parrots based on zoo, trade and conservation data
Source: R Soc Open Sci. 2026 Nov 11;13(11):261135. doi: 10.1098/rsos.261135 (PMC13750944; doi:10.1098/rsos.261135)
Supplement: Supplementary file 1 [file rsos.261135.zip › rsos.261135/rsos261135_si_002.docx]

# 1. Supplementary Material

## 1.1. Model diagnostics for GLMMs, GAMs, and GAMMs

Ex situ population dynamics of Brazilian parrots based on zoo, trade, and conservation data

Balazar et al.

# 2. Table S0: Summary of model diagnostics

This summary table presents the diagnostic outcomes for all models fitted in the study, along with the decision about whether each model was retained for inference or reported for descriptive purposes only. Detailed diagnostics are presented in Tables S1-S11.

Summary table S0. Model diagnostics outcomes and retention decisions.

| **Model** | **Assumptions_met** | **Retained_for** | **Notes** |
| --- | --- | --- | --- |
| GLMM (births) | Yes | Inference | Marginal dispersion deviation (p = 0.014), acceptable in practice for negative binomial models. |
| Global GAMs | No | Descriptive only | Intrinsic heterogeneity of NetChange_pc; see Tables S2-S4. |
| Genus-level GAMs | Yes (all 22 genera) | Inference | All 22 genera had at least one specification with adequate uniformity; see Tables S5-S7. |
| GAMM (sex ratio) | Partially | Inference with caution | Low R-squared (0.013) but significant smooth term for sex ratio (p = 0.011); see Table S8. |
| GAMM (trade) | Yes | Inference | Excellent fit (R-squared = 0.455); see Tables S9-S11. |

# 3. Supplementary Methods S1. Diagnostic procedures

All statistical models (GLMMs, GAMs, and GAMMs) were subjected to comprehensive diagnostic procedures to verify that model assumptions were adequately met. We used the following approaches:

### 3.1.1. S1.1. Simulation-based residual diagnostics (DHARMa package)

We used the DHARMa package (Hartig, 2022) to perform simulation-based residual diagnostics. DHARMa generates simulated residuals from the fitted model and compares the observed residual patterns to those expected under the assumed distribution. For each model, we tested:

(i) Uniformity: whether residuals follow a uniform distribution. A significant p-value indicates deviation from the expected pattern.

(ii) Dispersion: whether observed variance matches expected variance.

(iii) Outliers: number of observations with residuals more extreme than expected (tested via bootstrap, n = 1000).

(iv) Quantile deviations: whether residuals follow the expected distribution across quantiles.

Models were considered to have adequate residual structure when uniformity p > 0.05.

### 3.1.2. S1.2. Basis dimension assessment (gam.check, mgcv package)

For all GAMs and GAMMs, we used gam.check() from the mgcv package (Wood, 2011) to assess the adequacy of the basis dimension (k) for each smooth term. The k-index and its p-value indicate whether the chosen k is sufficient to capture the underlying curvature. Values of k-index > 0.90 were considered adequate. For random-effect smooths (bs = 're'), the k-index test is not defined (returns NA), which is expected behavior of the mgcv package.

### 3.1.3. S1.3. Concurvity assessment

Concurvity (the nonlinear analogue of collinearity) was assessed using concurvity() from the mgcv package, with full = TRUE to evaluate the worst-case scenario. Observed concurvity values > 0.80 indicate strong overlap among smooth terms. High concurvity is expected when multiple smooths share the same covariate (e.g., s(Year) and s(Year, Species)) and does not invalidate inference but may inflate uncertainty.

### 3.1.4. S1.4. Temporal autocorrelation

Because observations were repeated across years within species, we assessed temporal autocorrelation in residuals. The DHARMa function testTemporalAutocorrelation() requires balanced panels; because our data were unbalanced, this test failed in most cases. We therefore computed the mean autocorrelation function at lag-1 (ACF-1) across species (or genera) using residuals from the fitted models. Values close to zero indicate no systematic temporal autocorrelation.

### 3.1.5. S1.5. Model fit measures

For each model, we report adjusted R-squared, deviance explained (for GAMs), marginal and conditional R-squared (Nakagawa's R-squared, via performance package, for GLMMs), AIC, BIC, and REML score (for GAMs/GAMMs fitted with REML).

### 3.1.6. S1.6. Residual normality (for GAMMs with Gaussian family)

For the GAMM of trade, we additionally assessed normality of normalized residuals using Anderson-Darling, Shapiro-Wilk (on random subsamples of n = 500 and n = 5000 due to the n <= 5000 limit), and Kolmogorov-Smirnov tests, complemented by skewness, kurtosis, Q-Q plot, and histogram. Formal normality tests are highly sensitive at large sample sizes; we therefore complemented them with effect size measures and visual inspection.

### 3.1.7. S1.7. Sensitivity analysis

For the GAMM of trade, we tested alternative basis dimensions (k = 8, 12, 15). For genus-level GAMs, we tested two complementary specifications: continuous (scaled-t distribution, family = scat) and binary (binomial distribution, response = NetChange_pc > 0).

### 3.1.8. References

Hartig, F. (2022). DHARMa: Residual Diagnostics for Hierarchical (Multi-Level / Mixed) Regression Models.

Wood, S. N. (2011). Fast stable restricted maximum likelihood and marginal likelihood estimation of semiparametric generalized linear models. Journal of the Royal Statistical Society: Series B, 73(1), 3-36.

# 4. Table S1. GLMM for births - full diagnostics

Table S1. Full diagnostics for the negative binomial GLMM fitted to annual births.

| **Category** | **Metric** | **Value** |
| --- | --- | --- |
| **Model** | Family | nbinom2 (negative binomial) |
|  | Formula | Births ~ Year_c + IUCN + (1\|Species) + offset(log(Total)) |
|  | N | 1259 |
|  | Groups (Species) | 63 |
|  | Convergence | Code 0 (successful) |
|  | Singular fit | No |
| **Fit** | Marginal R2 | 0.275 |
|  | Conditional R2 | 0.409 |
|  | AIC | 5391.31 |
|  | BIC | 5437.55 |
|  | logLik | -2686.65 |
| **DHARMa** | Uniformity | p = 0.121 (OK) |
|  | Dispersion | p = 0.014 (marginal deviation) |
|  | Outliers | p = 0.820 (OK) |
|  | Zero inflation | Not applicable |
|  | Temporal autocorrelation | Not applicable (unbalanced panels) |
| **Collinearity** | Max VIF | 1.068 |
|  | Min Tolerance | 0.936 |
| **Key effects** | Year_c (IRR, p) | 0.813 (0.773-0.856), p < 0.001 |
|  | IUCN (all categories) | Not significant (all p > 0.15) |

### 4.1.1. Interpretation:

The GLMM showed adequate fit (conditional R2 = 0.409, marginal R2 = 0.275). Uniformity was acceptable (p = 0.121) and outliers were not detected (p = 0.820). Dispersion showed a marginal deviation (p = 0.014), which is common in negative binomial models with high overdispersion and does not invalidate inference. Zero-inflation diagnostics were not applicable because the negative binomial distribution accounts for excess zeros structurally. Temporal autocorrelation could not be tested within the current model specification. All VIF values were < 1.1, indicating no problematic collinearity. The model converged successfully with no evidence of singular fit.

### 4.1.2. Verdict:

Model assumptions were adequately met. The model was retained for inference.

# 5. Table S2. Global GAMs - fit comparison

Table S2. Fit comparison across three specifications of the global GAM for NetChange_pc.

| **Model** | **Family** | **N** | **Adjusted_R2** | **Deviance_expl** | **AIC** | **BIC** | **Converged** |
| --- | --- | --- | --- | --- | --- | --- | --- |
| M1 - Gaussian original | gaussian | 1259 | 0.016 | 2.91% | 372.14 | 485.94 | TRUE |
| M2 - scat original | scaled-t | 1259 | -0.005 | 1.75% | -834.29 | -700.87 | TRUE |
| M4 - scat without s(Year) | scaled-t | 1259 | -0.004 | 1.12% | -835.15 | -744.02 | TRUE |

### 5.1.1. Interpretation:

All global GAM specifications explained negligible variance (adjusted R2 <= 0.016), indicating that NetChange_pc is intrinsically heterogeneous across species. Removing the redundant s(Year) term (M4) marginally improved AIC (-0.86) and BIC (-43.15) but did not improve explanatory power. These models are reported for descriptive purposes only, and inference is based on genus-level models (Table S5).

### 5.1.2. Verdict:

Model assumptions were NOT adequately met. These models were retained for descriptive purposes only; inference is based on genus-level models (see Tables S5-S7).

# 6. Table S3. Global GAMs - DHARMa diagnostics

Table S3. DHARMa diagnostics for the three global GAM specifications.

| **Model** | **Test** | **Statistic** | **P_value** | **Result** |
| --- | --- | --- | --- | --- |
| M1 - Gaussian | Uniformity | 0.202 | 6.7e-45 | Fail |
| M1 - Gaussian | Dispersion | 0.987 | 0.762 | OK |
| M1 - Gaussian | Outliers | 26 | <0.001 | Fail |
| M1 - Gaussian | Quantiles | - | <0.001 | Fail |
| M2 - scat | Uniformity | 0.077 | 7.9e-07 | Fail |
| M2 - scat | Dispersion | 2.667 | 0.016 | Fail |
| M2 - scat | Outliers | 15 | <0.001 | Fail |
| M2 - scat | Quantiles | - | 2.2e-05 | Fail |
| M4 - scat no Year | Uniformity | 0.074 | 1.8e-06 | Fail |
| M4 - scat no Year | Dispersion | 2.631 | 0.016 | Fail |
| M4 - scat no Year | Outliers | 14 | <0.001 | Fail |
| M4 - scat no Year | Quantiles | - | <0.001 | Fail |

Autocorrelation (mean ACF lag-1): M1 = -0.055; M2 = -0.059; M4 = -0.051 (across 63 species).

### 6.1.1. Interpretation:

None of the global specifications met DHARMa assumptions. The failures are consistent across families (Gaussian, scaled-t) and specifications (with/without s(Year)), confirming that the lack of fit reflects intrinsic heterogeneity of the response variable rather than model misspecification.

### 6.1.2. Verdict:

Model assumptions were NOT adequately met for any specification. Inference from these models was not performed.

# 7. Table S4. Global GAMs - concurvity

Table S4. Observed concurvity for the global GAM specifications.

| **Model** | **Term** | **Concurvity_observed** |
| --- | --- | --- |
| **M1 - With s(Year)** | parametric | 0.992 |
|  | s(Year) | 1.000 |
|  | s(Year, Species) | 0.975 |
|  | s(Species) | 1.000 |
| **M4 - Without s(Year)** | parametric | 0.995 |
|  | s(Year, Species) | 0.975 |
|  | s(Species) | 1.000 |

### 7.1.1. AIC/BIC comparison:

With s(Year): AIC = 372.14, BIC = 485.94. Without s(Year): AIC = 371.77, BIC = 442.80.

### 7.1.2. Interpretation:

Concurvity between the parametric term and smooths is high (0.99) due to the intercept capturing the overall mean. The s(Year, Species) term shows high concurvity (0.975) with the parametric term because it inherently includes the year dimension. Removing the redundant s(Year) term (M4) did not reduce concurvity, confirming that high concurvity is a structural feature of the model specification, not a sign of misspecification.

### 7.1.3. Verdict:

Concurvity was high but structural; it did not compromise inference. Reported for transparency.

# 8. Table S5. Genus-level GAMs - fit comparison (scat vs binomial)

Table S5. Fit comparison across two complementary specifications for genus-level GAMs.

| **Genus** | **N** | **Species** | **R2_scat** | **R2_binom** | **Best_model** | **Best_R2** |
| --- | --- | --- | --- | --- | --- | --- |
| Pionopsitta | 21 | 1 | 0.333 | 0.019 | scat | 0.333 |
| Primolius | 63 | 3 | 0.282 | 0.093 | scat | 0.282 |
| Ara | 84 | 4 | 0.211 | 0.270 | binomial | 0.270 |
| Psittacara | 42 | 2 | 0.214 | 0.205 | scat | 0.214 |
| Cyanopsitta | 15 | 1 | 0.176 | 0.020 | scat | 0.176 |
| Myiopsitta | 21 | 1 | 0.134 | 0.040 | scat | 0.134 |
| Eupsittula | 58 | 3 | 0.082 | 0.131 | binomial | 0.131 |
| Aratinga | 105 | 5 | 0.009 | 0.106 | binomial | 0.106 |
| Diopsittaca | 21 | 1 | 0.012 | 0.096 | binomial | 0.096 |
| Pionus | 63 | 3 | -0.026 | 0.084 | binomial | 0.084 |
| Pyrrhura | 209 | 12 | -0.004 | 0.079 | binomial | 0.079 |
| Brotogeris | 100 | 5 | -0.010 | 0.065 | binomial | 0.065 |
| Orthopsittaca | 21 | 1 | 0.053 | -0.039 | scat | 0.053 |
| Deroptyus | 21 | 1 | -0.013 | 0.028 | binomial | 0.028 |
| Amazona | 226 | 11 | -0.014 | 0.025 | binomial | 0.025 |
| Forpus | 21 | 1 | -0.030 | 0.005 | binomial | 0.005 |
| Graydidascalus | 21 | 1 | 0.003 | -0.019 | scat | 0.003 |
| Alipiopsitta | 21 | 1 | -0.049 | -0.031 | - | - |
| Anodorhynchus | 42 | 2 | -0.034 | -0.009 | - | - |
| Guaruba | 21 | 1 | -0.069 | -0.042 | - | - |
| Pionites | 42 | 2 | -0.007 | -0.020 | - | - |
| Triclaria | 21 | 1 | -0.112 | -0.016 | - | - |

### 8.1.1. Interpretation:

Fourteen genera had at least one specification with adjusted R2 > 0 (moderate explanatory power). Nine genera had strong fit (R2 > 0.10), five had moderate fit (R2 = 0.05-0.10), and five showed low or negative R2, indicating the absence of a detectable temporal trend rather than model misspecification.

### 8.1.2. Verdict:

Model assumptions were adequately met for 14 of 22 genera. For the remaining eight genera, models were retained for descriptive purposes only, as low R2 reflects the absence of a temporal signal rather than model failure.

# 9. Table S6. Genus-level GAMs - DHARMa uniformity

Table S6. DHARMa uniformity p-values for continuous (scat) and binary (binomial) genus-level GAM specifications.

| **Genus** | **Uniformity_scat** | **Uniformity_binom** | **At_least_one_OK** |
| --- | --- | --- | --- |
| Alipiopsitta | 0.962 | 0.819 | Yes |
| Amazona | 0.484 | 0.851 | Yes |
| Anodorhynchus | 0.422 | 0.704 | Yes |
| Ara | 0.904 | 0.616 | Yes |
| Aratinga | 0.797 | 0.312 | Yes |
| Brotogeris | 0.003 | 0.929 | Yes (binom) |
| Cyanopsitta | 0.927 | 0.965 | Yes |
| Deroptyus | 0.964 | 0.196 | Yes |
| Diopsittaca | 0.764 | 0.605 | Yes |
| Eupsittula | 0.713 | 0.450 | Yes |
| Forpus | 0.627 | 0.920 | Yes |
| Graydidascalus | 0.333 | 0.652 | Yes |
| Guaruba | 0.807 | 0.783 | Yes |
| Myiopsitta | 0.997 | 0.881 | Yes |
| Orthopsittaca | 0.897 | 0.291 | Yes |
| Pionites | 0.733 | 0.827 | Yes |
| Pionopsitta | 0.945 | 0.865 | Yes |
| Pionus | 0.924 | 0.782 | Yes |
| Primolius | 0.946 | 0.346 | Yes |
| Psittacara | 0.922 | 0.824 | Yes |
| Pyrrhura | 0.308 | 0.308 | Yes (borderline) |
| Triclaria | 0.600 | 0.950 | Yes |

### 9.1.1. Interpretation:

All 22 genera had at least one specification with adequate residual uniformity (p > 0.05). The binary specification resolved uniformity failures observed in the continuous specification for Brotogeris (p = 0.003 -> 0.929), demonstrating the value of testing complementary specifications.

### 9.1.2. Verdict:

All 22 genera had at least one specification with adequate residual uniformity (p > 0.05). Model assumptions were adequately met for every genus.

# 10. Table S7. Genus-level GAMs - classification by quality

Table S7. Classification of genus-level GAMs based on fit and diagnostic quality.

| **Category** | **Definition** | **N_genera** | **Genera** |
| --- | --- | --- | --- |
| Strong fit | Best R2 > 0.10, uniformity p > 0.05 | 9 | Pionopsitta, Primolius, Ara, Psittacara, Cyanopsitta, Myiopsitta, Eupsittula, Aratinga, Diopsittaca |
| Moderate fit | R2 = 0.05-0.10, uniformity p > 0.05 | 4 | Pionus, Pyrrhura, Brotogeris, Orthopsittaca |
| Weak fit | R2 < 0.05, uniformity p > 0.05 | 4 | Deroptyus, Amazona, Forpus, Graydidascalus |
| No detectable trend | R2 < 0, uniformity p > 0.05 | 5 | Alipiopsitta, Anodorhynchus, Guaruba, Pionites, Triclaria |

### 10.1.1. Interpretation:

Genus-level models were classified into four quality tiers based on adjusted R2 and DHARMa uniformity. Strong and Moderate categories (13 genera) were used for inference; Weak and No-trend categories (9 genera) were reported for completeness, as low R2 in these cases reflects the absence of a detectable temporal signal rather than model failure.

### 10.1.2. Verdict:

Genus-level models were classified into four quality tiers. Strong and Moderate categories (13 genera) were used for inference; Weak and No-trend categories (9 genera) were reported for completeness.

# 11. Table S8. GAMM for sex ratio - diagnostics

Table S8. Diagnostics for the GAMM relating sex ratio and IUCN to per capita population change.

| **Metric** | **Value** |
| --- | --- |
| N | 1197 |
| Adjusted R2 | 0.0129 |
| Deviance explained | 1.98% |
| s(SexRatio) edf | 3.307 |
| s(SexRatio) F | 4.264 |
| s(SexRatio) p-value | 0.011 |
| AIC | 10961.9 |
| REML score | 5462.6 |
| Converged | TRUE |
| DHARMa Uniformity | p = 1.0e-30 (Fail) |
| DHARMa Dispersion | p = 0.896 (OK) |
| DHARMa Outliers | 29 outliers (Fail) |
| DHARMa Quantiles | p < 0.001 (Fail) |
| ACF lag-1 (mean) | -0.018 (63 genera) |
| Concurvity (observed) | 0.011 |

### 11.1.1. Interpretation:

The model confirmed a statistically significant non-linear effect of sex ratio on per capita population change (s(SexRatio), edf = 3.31, F = 4.26, p = 0.011), consistent with a modest optimum near balanced sex ratios. However, overall fit was low (R2 = 0.013), and DHARMa diagnostics indicated non-uniformity and outliers, reflecting the heterogeneity of the response variable.

### 11.1.2. Verdict:

Model assumptions were only partially met. The smooth term for sex ratio was statistically significant, but low R2 and residual non-uniformity reflect intrinsic heterogeneity of the response variable. Results were interpreted with caution.

# 12. Table S9. GAMM for trade - diagnostics

Table S9. Full diagnostics for the trade GAMM.

| **Category** | **Metric** | **Value** |
| --- | --- | --- |
| **Fit** | N | 12228 |
|  | Adjusted R2 | 0.455 |
|  | AIC (LME) | 41650.5 |
|  | BIC (LME) | 41887.6 |
|  | logLik | -20793.2 |
|  | Converged | TRUE |
| **s(year)** | edf | 10.05 |
|  | F | 5.933 |
|  | p-value | < 2e-16 |
|  | k-index | - |
| **gam.check** | k-index (s(year)) | 0.99 |
|  | p-value | 0.69 |
| **Concurvity** | parametric | 0.973 |
|  | s(year) | 0.011 |
| **ACF lag-1** | Mean ACF lag-1 | 0.033 |
|  | N groups | 56 |

### 12.1.1. Interpretation:

The trade GAMM showed excellent fit (adjusted R2 = 0.455). The smooth term for year was highly significant (edf = 10.05, F = 5.93, p < 0.001), and the basis dimension was adequate (k-index = 0.99, p = 0.69). Concurvity between the parametric intercept and the smooth term was high (0.973), which is expected in GAMMs with a global intercept and a smooth term, and does not affect inference.

### 12.1.2. Verdict:

Model assumptions were adequately met. The model was retained for inference.

# 13. Table S10. GAMM for trade - normality tests

Table S10. Normality diagnostics for standardized residuals of the trade GAMM.

| **Metric** | **Value** |
| --- | --- |
| Mean | 0.000 |
| SD | 0.998 |
| Skewness | 0.437 |
| Excess kurtosis | 0.921 |
| Minimum | -3.883 |
| Q1 | -0.677 |
| Median | -0.034 |
| Q3 | 0.594 |
| Maximum | 5.493 |

Formal normality tests.

| **Test** | **Statistic** | **P_value** | **N** |
| --- | --- | --- | --- |
| Anderson-Darling | 20.471 | 3.7e-24 | 12228 |
| Shapiro-Wilk (subsample) | 0.988 | 5.1e-20 | 5000 |
| Shapiro-Wilk (n=500) | 0.989 | 7.8e-04 | 500 |
| Kolmogorov-Smirnov | 0.028 | 1.7e-08 | 12228 |

### 13.1.1. Interpretation:

Standardized residuals had mean zero, unit variance, and small departures from normality (skewness = 0.44, excess kurtosis = 0.92). Formal normality tests rejected the null hypothesis of normality, but this is expected at N = 12,228 due to high statistical power. Effect sizes indicate that departures were minor and would not affect inference in linear mixed models with large samples.

### 13.1.2. Verdict:

Departures from normality were negligible in effect size. Model assumptions were adequately met in practice for inference in linear mixed models with large samples.

# 14. Table S11. GAMM for trade - sensitivity analysis

Table S11. Sensitivity analysis of the basis dimension (k) for the smooth term of year in the trade GAMM.

| **k** | **AIC** | **BIC** | **Adjusted_R2** | **EDF_year** |
| --- | --- | --- | --- | --- |
| 8 | 41671.62 | 41908.79 | 0.454 | 5.73 |
| 12 (original) | 41650.48 | 41887.64 | 0.455 | 10.05 |
| 15 | 41650.54 | 41887.71 | 0.455 | 11.74 |

### 14.1.1. Interpretation:

The choice of basis dimension did not affect conclusions. Adjusted R2 ranged from 0.454 to 0.455 across k = 8, 12, 15. The effective degrees of freedom (edf = 10.05 at k = 12) was well below the maximum, indicating adequate flexibility without overfitting. k = 12 was selected as the optimal balance.

### 14.1.2. Verdict:

Conclusions were robust to the choice of basis dimension. No evidence of overfitting or underfitting.

# 15. Table S12. Species-specific GAM results for 59 Brazilian parrot species.

Species-specific GAMs were fitted with the model structure NetChange_pc ~ s(Year, k = 8), family = scat, method = REML. Species with fewer than 5 observations or fewer than 5 unique values of NetChange_pc were not fitted (see Table S13 for details). Models are ordered by p-value of the smooth term for year. Significance codes: *** p < 0.001; ** p < 0.01; * p < 0.05; ns = not significant. All species-specific models met DHARMa residual uniformity assumptions (uniformity p > 0.05), indicating that model assumptions were adequately met.

| **Species** | **Common name** | **N** | **Adjusted R²** | **Deviance expl.** | **EDF** | **Chi.sq** | **p-value** | **Sig.** | **Uniformity p** |
| --- | --- | --- | --- | --- | --- | --- | --- | --- | --- |
| Ara macao | Scarlet macaw | 21 | 0.615 | 0.669 | 2.770 | 33.187 | 0.0000 | *** | 0.973 |
| Ara ararauna | Blue-and-yellow macaw | 21 | 0.496 | 0.554 | 2.302 | 20.014 | 0.0002 | *** | 0.573 |
| Primolius auricollis | Golden-collared macaw | 21 | 0.358 | 0.390 | 1.000 | 12.137 | 0.0005 | *** | 0.982 |
| Ara chloropterus | Red-and-green macaw | 21 | 0.457 | 0.553 | 3.528 | 16.994 | 0.0024 | ** | 0.957 |
| Pionopsitta pileata | Pileated parrot | 21 | 0.333 | 0.372 | 1.176 | 11.815 | 0.0025 | ** | 0.945 |
| Amazona amazonica | Orange-winged Amazon parrot | 21 | 0.179 | 0.220 | 1.000 | 5.354 | 0.0207 | * | 0.984 |
| Aratinga nenday | Nanday parakeet | 21 | 0.177 | 0.218 | 1.000 | 5.296 | 0.0214 | * | 0.754 |
| Aratinga solstitialis | Sun conure | 21 | 0.176 | 0.217 | 1.000 | 5.264 | 0.0218 | * | 0.718 |
| Anodorhynchus hyacinthinus | Hyacinth macaw | 21 | -0.092 | 0.118 | 1.000 | 4.746 | 0.0294 | * | 0.629 |
| Aratinga weddellii | Dusk-headed parakeet | 21 | 0.255 | 0.325 | 2.266 | 7.767 | 0.0425 | * | 0.907 |
| Eupsittula pertinax | Brown-throated parakeet | 21 | 0.209 | 0.271 | 1.559 | 5.341 | 0.0469 | * | 0.997 |
| Graydidascalus brachyurus | Short-tailed parrot | 21 | 0.003 | 0.078 | 1.000 | 3.890 | 0.0486 | * | 0.333 |
| Primolius couloni | Blue-headed macaw | 21 | 0.124 | 0.166 | 1.000 | 3.882 | 0.0488 | * | 0.885 |
| Pyrrhura griseipectus | Grey-breasted parakeet | 21 | 0.212 | 0.299 | 2.198 | 5.523 | 0.0905 | . | 0.845 |
| Ara severus | Chestnut-fronted macaw | 21 | 0.077 | 0.123 | 1.000 | 2.668 | 0.1024 | ns | 0.617 |
| Eupsittula aurea | Peach-fronted parakeet | 21 | 0.085 | 0.109 | 1.000 | 2.643 | 0.1040 | ns | 0.799 |
| Pyrrhura roseifrons | Rose-fronted parakeet | 21 | -0.117 | 0.256 | 3.133 | 7.017 | 0.1271 | ns | 0.847 |
| Amazona dufresniana | Blue-cheeked Amazon parrot | 16 | 0.078 | 0.140 | 1.000 | 2.272 | 0.1318 | ns | 0.992 |
| Brotogeris cyanoptera | Cobalt-winged parakeet | 21 | -0.060 | 0.047 | 1.000 | 2.184 | 0.1395 | ns | 0.987 |
| Orthopsittaca manilatus | Red-bellied macaw | 21 | 0.053 | 0.101 | 1.000 | 2.127 | 0.1448 | ns | 0.897 |
| Myiopsitta monachus | Monk parakeet | 21 | 0.134 | 0.216 | 1.897 | 4.371 | 0.1792 | ns | 0.997 |
| Amazona vinacea | Vinaceous-breasted Amazon parrot | 21 | 0.038 | 0.086 | 1.000 | 1.780 | 0.1821 | ns | 0.998 |
| Triclaria malachitacea | Blue-bellied parrot | 21 | -0.056 | 0.069 | 1.100 | 2.293 | 0.1863 | ns | 0.639 |
| Amazona ochrocephala | Yellow-crowned Amazon parrot | 21 | 0.035 | 0.083 | 1.000 | 1.727 | 0.1889 | ns | 0.913 |
| Psittacara acuticaudatus | Blue-crowned parakeet | 21 | 0.004 | 0.073 | 1.000 | 1.647 | 0.1994 | ns | 0.983 |
| Amazona pretei |  | 21 | 0.020 | 0.048 | 1.000 | 1.635 | 0.2011 | ns | 0.927 |
| Pionites leucogaster | White-bellied parrot | 21 | 0.024 | 0.073 | 1.000 | 1.492 | 0.2219 | ns | 0.973 |
| Primolius maracana | Blue-winged macaw | 21 | 0.082 | 0.211 | 2.657 | 4.605 | 0.2273 | ns | 0.769 |
| Diopsittaca nobilis | Red-shouldered macaw | 21 | 0.012 | 0.036 | 1.000 | 1.451 | 0.2284 | ns | 0.764 |
| Cyanopsitta spixii | Spix's macaw | 15 | 0.072 | 0.063 | 1.000 | 1.400 | 0.2368 | ns | 0.932 |
| Amazona rhodocorytha | Red-browed Amazon parrot | 21 | 0.128 | 0.219 | 2.086 | 3.059 | 0.2509 | ns | 0.609 |
| Eupsittula cactorum | Cactus parakeet | 16 | 0.133 | 0.193 | 3.010 | 5.894 | 0.2859 | ns | 0.635 |
| Pionus menstruus | Blue-headed parrot | 21 | 0.005 | 0.054 | 1.000 | 1.092 | 0.2960 | ns | 0.856 |
| Pionus maximiliani | Scaly-headed parrot | 21 | -0.069 | 0.035 | 1.000 | 1.001 | 0.3171 | ns | 0.996 |
| Forpus passerius |  | 21 | -0.030 | 0.022 | 1.000 | 0.916 | 0.3386 | ns | 0.627 |
| Amazona farinosa | Mealy Amazon parrot | 21 | -0.005 | 0.045 | 1.000 | 0.900 | 0.3429 | ns | 0.784 |
| Amazona autumnalis | Red-lored Amazon parrot | 21 | -0.071 | 0.029 | 1.000 | 0.882 | 0.3478 | ns | 0.596 |
| Deroptyus accipitrinus | Red-fan parrot | 21 | -0.013 | 0.034 | 1.000 | 0.841 | 0.3591 | ns | 0.964 |
| Brotogeris chiriri | Yellow-chevroned parakeet | 21 | 0.001 | 0.034 | 1.000 | 0.724 | 0.3948 | ns | 0.535 |
| Brotogeris versicolurus | White-winged parakeet | 19 | -0.052 | 0.054 | 1.740 | 2.060 | 0.4273 | ns | 0.210 |
| Amazona brasiliensis | Red-tailed Amazon parrot | 21 | -0.010 | 0.074 | 1.753 | 1.710 | 0.4406 | ns | 0.913 |
| Pyrrhura cruentata | Ochre-marked parakeet | 21 | -0.028 | 0.023 | 1.000 | 0.453 | 0.5010 | ns | 0.541 |
| Pyrrhura rupicola | Black-capped parakeet | 20 | -0.001 | 0.011 | 1.000 | 0.338 | 0.5608 | ns | 0.434 |
| Pyrrhura molinae | Green-cheeked parakeet | 21 | -0.097 | 0.069 | 1.732 | 1.451 | 0.5831 | ns | 0.996 |
| Brotogeris tirica | Plain parakeet | 18 | -0.046 | 0.016 | 1.000 | 0.254 | 0.6144 | ns | 0.550 |
| Pyrrhura perlata | Crimson-bellied parakeet | 21 | -0.040 | 0.012 | 1.000 | 0.231 | 0.6310 | ns | 0.879 |
| Pionus fuscus | Dusky parrot | 21 | -0.042 | 0.011 | 1.000 | 0.203 | 0.6524 | ns | 0.919 |
| Anodorhynchus leari | Lear's macaw | 21 | -0.045 | 0.036 | 1.392 | 0.355 | 0.7136 | ns | 0.449 |
| Pionites melanocephalus | Black-headed parrot | 21 | -0.015 | 0.059 | 1.447 | 0.404 | 0.7398 | ns | 0.642 |
| Guaruba guarouba | Golden parakeet | 21 | -0.021 | 0.044 | 1.271 | 0.205 | 0.7452 | ns | 0.834 |
| Pyrrhura frontalis | Maroon-bellied parakeet | 21 | -0.048 | 0.004 | 1.000 | 0.086 | 0.7699 | ns | 0.893 |
| Pyrrhura picta | Painted parakeet | 21 | -0.043 | 0.003 | 1.011 | 0.078 | 0.7893 | ns | 0.801 |
| Alipiopsitta xanthops | Yellow-faced parrot | 21 | -0.049 | 0.003 | 1.000 | 0.059 | 0.8081 | ns | 0.962 |
| Aratinga jandaya | Jandaya parakeet | 21 | -0.039 | 0.012 | 1.078 | 0.074 | 0.8278 | ns | 0.837 |
| Pyrrhura leucotis | White-eared parakeet | 21 | -0.064 | 0.001 | 1.000 | 0.029 | 0.8651 | ns | 0.831 |
| Aratinga auricapillus | Golden-capped parakeet | 21 | -0.051 | 0.001 | 1.000 | 0.027 | 0.8706 | ns | 0.565 |
| Psittacara leucophthalmus | White-eyed parakeet | 21 | -0.048 | 0.001 | 1.000 | 0.026 | 0.8716 | ns | 0.759 |
| Amazona aestiva | Blue-fronted Amazon parrot | 21 | -0.053 | 0.000 | 1.000 | 0.003 | 0.9601 | ns | 0.665 |
| Amazona festiva | Festive Amazon parrot | 21 | -0.051 | 0.000 | 1.003 | 0.000 | 0.9978 | ns | 0.645 |

# 16. Supplementary Figures - Captions

### 16.1.1. Figure S1. DHARMa residual diagnostics for the three global GAM specifications of NetChange_pc.

DHARMa simulated residual diagnostics for the three global GAM specifications of per capita population change (NetChange_pc). (A) M1 - Gaussian original family. (B) M2 - scaled-t (scat) family with the same model structure. (C) M4 - scaled-t family without the redundant s(Year) smooth term. Each panel displays the Q-Q plot of simulated residuals, residuals vs. predicted values, observed vs. expected quantiles, and residuals vs. each predictor. None of the three specifications met DHARMa uniformity assumptions (p < 0.001 in all cases), confirming that the low explanatory power reflects the intrinsic heterogeneity of NetChange_pc across species rather than model misspecification.

### 16.1.2. Figure S2. DHARMa residual diagnostics for genus-level GAMs with strong or acceptable fit.

DHARMa simulated residual diagnostics for the eight genus-level GAMs classified as having strong (R2 > 0.10) or acceptable (R2 = 0.05-0.10) fit. (A) Pionopsitta (scaled-t specification; uniformity p = 0.945). (B) Primolius (scaled-t; p = 0.946). (C) Ara (binomial; p = 0.616). (D) Psittacara (scaled-t; p = 0.922). (E) Cyanopsitta (scaled-t; p = 0.927). (F) Myiopsitta (scaled-t; p = 0.997). (G) Eupsittula (binomial; p = 0.450). (H) Aratinga (binomial; p = 0.312). For each genus, the specification with the best combination of adjusted R2 and residual uniformity is shown. All specifications met DHARMa uniformity assumptions (p > 0.05).

### 16.1.3. Figure S3. DHARMa residual diagnostics for genus-level GAMs with weak fit or no detectable trend.

DHARMa simulated residual diagnostics for the fourteen genus-level GAMs classified as having weak fit (R2 < 0.05) or no detectable temporal trend (R2 < 0). (A) Diopsittaca (binomial; uniformity p = 0.605). (B) Pionus (binomial; p = 0.782). (C) Pyrrhura (binomial; p = 0.308). (D) Brotogeris (binomial; p = 0.929). (E) Orthopsittaca (scaled-t; p = 0.897). (F) Deroptyus (binomial; p = 0.196). (G) Amazona (binomial; p = 0.851). (H) Forpus (binomial; p = 0.920). (I) Graydidascalus (scaled-t; p = 0.333). (J) Alipiopsitta (binomial; p = 0.819). (K) Anodorhynchus (binomial; p = 0.704). (L) Guaruba (binomial; p = 0.783). (M) Pionites (binomial; p = 0.827). (N) Triclaria (binomial; p = 0.950). For each genus, the specification with the best combination of adjusted R2 and residual uniformity is shown. All specifications met DHARMa uniformity assumptions (p > 0.05). Low or negative R2 in these panels reflects the absence of a detectable temporal trend rather than model misspecification.

### 16.1.4. Figure S4. DHARMa residual diagnostics for the GAMM relating sex ratio and IUCN to NetChange_pc.

DHARMa simulated residual diagnostics for the generalized additive mixed model (GAMM) relating sex ratio (male/total) and IUCN threat category to per capita population change (NetChange_pc), with genus as a random effect. The model had adequate dispersion (p = 0.896) but non-uniform residuals (p < 0.001) and 29 outliers. The smooth term for sex ratio was statistically significant (edf = 3.31, F = 4.26, p = 0.011), confirming a non-linear relationship, but overall explanatory power was low (adjusted R2 = 0.013), reflecting the heterogeneity of the response variable.

### 16.1.5. Figure S5. Residual diagnostics for the trade GAMM.

Residual diagnostics for the generalized additive mixed model (GAMM) fitted to international trade volume (log_trade ~ threat_level + log_captive + importer_region + exporter_region + purpose + source + s(year, k = 12), with taxon as a random effect; N = 12,228). (A) Normalized residuals vs. fitted values. (B) Q-Q plot of standardized residuals. (C) Autocorrelation function (ACF) of residuals; no systematic temporal autocorrelation was detected (mean ACF lag-1 = 0.033). (D) Normalized residuals across years, showing no temporal trend. The model showed excellent fit (adjusted R2 = 0.455) with a highly significant smooth term for year (edf = 10.05, F = 5.93, p < 2 x 10^-16) and adequate basis dimension (k-index = 0.99, p = 0.69).

### 16.1.6. Figure S6. Normality diagnostics for the standardized residuals of the trade GAMM.

Normality diagnostics for the standardized residuals of the trade GAMM (N = 12,228). (A) Histogram of standardized residuals overlaid with the normal density curve (solid red line). Residuals were approximately symmetric (skewness = 0.44) with small excess kurtosis (0.92) and unit variance (SD = 0.998). Although formal normality tests (Anderson-Darling, Shapiro-Wilk, Kolmogorov-Smirnov) rejected the null hypothesis of normality, this is expected at large sample sizes due to high statistical power. The effect size measures and visual inspection indicate that departures from normality were negligible and would not affect inference in linear mixed models.

Figure S1

Figure S2

Figure S3

Figure S4

Figure S5

Figure S6
