# Supplementary material for: Ex situ population dynamics of Brazilian parrots based on zoo, trade and conservation data
Source: R Soc Open Sci. 2026 Nov 11;13(11):261135. doi: 10.1098/rsos.261135 (PMC13750944; doi:10.1098/rsos.261135)
Supplement: Supplementary file 1 [file rsos.261135.zip › rsos.261135/rsos261135_si_003.docx]

**Supplementary Material 3.** Descriptive statistics of birth, mortality, acquisition, and net per capita population change (NetChange_pc) rates for Psittacidae species, presented as mean ± standard deviation (SD). IUCN categories: Least Concern (LC); Near Threatened (NT); Vulnerable (VU); Endangered (EN); Critically Endangered (CR); Extinct in the Wild (EW).

| **Species** | **IUCN** | **Birth** | **Mortality** | **Acquisition** | **NetChange** |
| --- | --- | --- | --- | --- | --- |
| *Alipiopsitta xanthops* | NT | 6.50 ± 6.57 | 6.25 ± 4.79 | 4.82 ± 6.78 | 0.74 ± 13.97 |
| *Amazona aestiva* | NT | 1.15 ± 0.28 | 6.29 ± 2.16 | 10.85 ± 2.11 | 1.13 ± 2.42 |
| *Amazona aestiva* | LC | 4.56 ± 3.15 | 5.29 ± 1.44 | 9.36 ± 4.91 | 2.33 ± 4.36 |
| *Amazona amazonica* | LC | 3.33 ± 2.11 | 6.64 ± 2.11 | 8.71 ± 3.47 | 1.69 ± 4.94 |
| *Amazona autumnalis* | LC | 1.62 ± 1.57 | 5.37 ± 3.45 | 10.85 ± 5.93 | 3.80 ± 5.73 |
| *Amazona brasiliensis* | VU | 4.13 ± 3.47 | 5.53 ± 2.96 | 5.02 ± 5.56 | 1.64 ± 7.09 |
| *Amazona brasiliensis* | NT | 5.23 ± 3.30 | 9.69 ± 4.16 | 4.93 ± 9.05 | -2.84 ± 13.69 |
| *Amazona dufresniana* | NT | 0.00 ± 0.00 | 14.44 ± 28.78 | 36.67 ± 71.88 | 7.29 ± 50.91 |
| *Amazona farinosa* | NT | 0.66 ± 0.79 | 6.92 ± 3.26 | 8.01 ± 3.40 | -0.05 ± 6.37 |
| *Amazona farinosa* | LC | 3.27 ± 4.33 | 5.84 ± 3.63 | 9.63 ± 4.45 | 3.14 ± 6.87 |
| *Amazona festiva* | NT | 9.08 ± 2.52 | 8.71 ± 3.24 | 2.22 ± 3.45 | -6.07 ± 4.44 |
| *Amazona festiva* | LC | 7.45 ± 16.29 | 5.33 ± 3.69 | 5.51 ± 4.30 | 2.79 ± 11.77 |
| *Amazona ochrocephala* | LC | 1.01 ± 0.91 | 5.31 ± 2.55 | 11.29 ± 5.34 | 2.74 ± 5.84 |
| *Amazona pretei* | VU | 45.45 ± 178.49 | 15.52 ± 18.75 | 15.64 ± 26.10 | -16.35 ± 74.57 |
| *Amazona rhodocorytha* | EN | 2.62 ± 8.05 | 6.16 ± 5.90 | 15.86 ± 16.78 | 5.17 ± 15.67 |
| *Amazona rhodocorytha* | VU | 0.67 ± 1.23 | 4.62 ± 3.02 | 6.26 ± 5.52 | -4.16 ± 10.53 |
| *Amazona vinacea* | EN | 9.83 ± 3.94 | 14.26 ± 6.24 | 10.04 ± 7.83 | 1.25 ± 10.37 |
| *Amazona vinacea* | VU | 10.02 ± 6.97 | 10.04 ± 3.09 | 6.92 ± 7.50 | 7.31 ± 13.66 |
| *Anodorhynchus hyacinthinus* | EN | 4.73 ± 1.20 | 5.65 ± 0.57 | 3.85 ± 0.94 | 1.54 ± 1.16 |
| *Anodorhynchus hyacinthinus* | VU | 4.69 ± 1.86 | 6.08 ± 1.71 | 3.70 ± 4.69 | 0.64 ± 4.47 |
| *Anodorhynchus leari* | CR | 4.21 ± 5.89 | 0.00 ± 0.00 | 14.06 ± 28.12 | 1.99 ± 30.46 |
| *Anodorhynchus leari* | EN | 5.90 ± 4.73 | 1.53 ± 1.81 | 11.53 ± 28.94 | 9.51 ± 14.45 |
| *Ara ararauna* | LC | 3.50 ± 1.32 | 4.02 ± 0.95 | 6.13 ± 1.43 | 2.13 ± 2.25 |
| *Ara chloropterus* | LC | 3.67 ± 0.84 | 4.46 ± 1.19 | 4.96 ± 1.19 | 1.02 ± 2.02 |
| *Ara macao* | LC | 5.48 ± 2.60 | 3.91 ± 1.69 | 2.68 ± 1.26 | 2.04 ± 4.37 |
| *Ara severus* | LC | 3.80 ± 3.86 | 6.30 ± 3.23 | 8.87 ± 5.31 | 1.40 ± 6.83 |
| *Aratinga auricapillus* | NT | 3.29 ± 5.05 | 6.12 ± 4.57 | 4.30 ± 10.00 | -4.79 ± 20.26 |
| *Aratinga auricapillus* | LC | 5.26 ± 9.12 | 13.42 ± 18.87 | 1.67 ± 2.89 | -8.15 ± 23.27 |
| *Aratinga jandaya* | LC | 4.69 ± 2.54 | 9.68 ± 4.54 | 10.39 ± 6.53 | 0.35 ± 11.31 |
| *Aratinga nenday* | LC | 4.88 ± 2.87 | 7.62 ± 3.60 | 3.38 ± 2.85 | -0.89 ± 5.03 |
| *Aratinga solstitialis* | EN | 12.08 ± 2.67 | 12.69 ± 1.69 | 7.51 ± 4.01 | 2.80 ± 5.16 |
| *Aratinga solstitialis* | LC | 13.06 ± 2.24 | 11.28 ± 0.99 | 9.30 ± 3.34 | 6.39 ± 3.27 |
| *Aratinga weddellii* | LC | 1.97 ± 5.47 | 4.21 ± 5.29 | 10.64 ± 11.14 | 1.97 ± 15.70 |
| *Brotogeris chiriri* | LC | 1.25 ± 5.59 | 19.43 ± 25.14 | 33.84 ± 47.44 | 6.00 ± 28.33 |
| *Brotogeris cyanoptera* | LC | 0.00 ± 0.00 | 25.25 ± 38.66 | 64.64 ± 94.48 | -23.58 ± 106.32 |
| *Brotogeris sanctithomae* | LC | 0.00 ± 0.00 | 2.25 ± 6.97 | 3.33 ± 14.91 | -0.87 ± 12.88 |
| *Brotogeris tirica* | LC | 6.00 ± 24.23 | 38.56 ± 96.02 | 194.23 ± 650.34 | 10.81 ± 60.54 |
| *Brotogeris versicolurus* | LC | 0.00 ± 0.00 | 10.51 ± 25.45 | 110.89 ± 277.81 | 14.84 ± 47.11 |
| *Cyanopsitta spixii* | EW | 0.00 ± 0.00 | 5.23 ± 6.90 | 88.67 ± 102.35 | 21.84 ± 31.95 |
| *Cyanopsitta spixii* | CR | 0.00 ± 0.00 | 0.00 ± 0.00 | 22.92 ± 52.66 | 0.66 ± 59.79 |
| *Deroptyus accipitrinus* | LC | 2.99 ± 2.56 | 6.63 ± 3.07 | 5.90 ± 5.10 | -1.75 ± 6.48 |
| *Diopsittaca nobilis* | LC | 10.58 ± 10.98 | 8.60 ± 4.56 | 5.78 ± 5.67 | 0.82 ± 14.07 |
| *Eupsittula aurea* | LC | 4.48 ± 5.45 | 9.51 ± 8.71 | 16.00 ± 20.40 | 4.22 ± 16.10 |
| *Eupsittula cactorum* | LC | 0.00 ± 0.00 | 9.67 ± 26.22 | 30.00 ± 84.09 | 9.93 ± 33.69 |
| *Eupsittula pertinax* | LC | 8.31 ± 13.42 | 14.73 ± 8.75 | 22.16 ± 25.29 | 6.93 ± 17.62 |
| *Forpus passerius* | LC | 2.18 ± 5.16 | 7.52 ± 10.30 | 129.06 ± 558.16 | -3.71 ± 34.43 |
| *Graydidascalus brachyurus* | LC | 109.37 ± 468.68 | 38.51 ± 132.70 | 0.71 ± 3.19 | -0.75 ± 23.85 |
| *Guaruba guarouba* | EN | 6.66 ± 3.80 | 8.84 ± 2.24 | 5.16 ± 2.63 | 1.65 ± 4.01 |
| *Guaruba guarouba* | VU | 7.49 ± 3.15 | 10.09 ± 1.59 | 4.87 ± 2.85 | 0.72 ± 3.82 |
| *Myiopsitta monachus* | LC | 10.29 ± 3.71 | 12.30 ± 3.12 | 6.18 ± 3.61 | 0.78 ± 5.84 |
| *Orthopsittaca manilatus* | LC | 1.94 ± 4.42 | 14.45 ± 10.18 | 20.54 ± 24.84 | 1.12 ± 22.08 |
| *Pionites leucogaster* | EN | 15.71 ± 8.32 | 11.88 ± 6.54 | 13.00 ± 11.64 | 6.31 ± 10.97 |
| *Pionites leucogaster* | VU | 11.86 ± 5.18 | 15.22 ± 5.30 | 10.18 ± 5.12 | 1.06 ± 5.45 |
| *Pionites leucogaster* | LC | 9.17 ± 6.77 | 9.69 ± 5.14 | 5.86 ± 3.82 | -1.26 ± 11.72 |
| *Pionites melanocephalus* | LC | 4.89 ± 3.39 | 10.24 ± 5.34 | 14.04 ± 10.78 | 0.77 ± 11.14 |
| *Pionopsitta pileata* | LC | 6.08 ± 18.14 | 17.92 ± 29.18 | 15.74 ± 18.64 | -1.29 ± 18.31 |
| *Pionus fuscus* | LC | 28.22 ± 62.88 | 14.10 ± 14.26 | 11.76 ± 15.52 | 0.69 ± 29.10 |
| *Pionus maximiliani* | LC | 4.23 ± 5.41 | 5.18 ± 5.31 | 10.25 ± 8.18 | 6.23 ± 8.50 |
| *Pionus menstruus* | LC | 3.15 ± 3.85 | 9.13 ± 4.24 | 14.61 ± 7.93 | 2.36 ± 8.99 |
| *Primolius auricollis* | LC | 3.45 ± 4.93 | 9.38 ± 6.78 | 6.44 ± 6.06 | -4.51 ± 10.38 |
| *Primolius couloni* | EN | 17.12 ± 4.04 | 7.49 ± 1.47 | 3.11 ± 3.94 | 5.16 ± 10.21 |
| *Primolius couloni* | VU | 12.11 ± 4.03 | 6.67 ± 2.92 | 2.77 ± 2.66 | 1.90 ± 6.04 |
| *Primolius couloni* | NT | NaN ± NA | NaN ± NA | NaN ± NA | 23.08 ± NA |
| *Primolius maracana* | NT | 6.39 ± 3.84 | 5.39 ± 3.27 | 5.92 ± 3.93 | 3.70 ± 7.42 |
| *Psittacara acuticaudatus* | LC | 3.25 ± 2.40 | 7.87 ± 5.03 | 5.82 ± 5.23 | -2.10 ± 7.43 |
| *Psittacara leucophthalmus* | LC | 3.07 ± 3.76 | 3.62 ± 3.48 | 14.81 ± 17.25 | 9.49 ± 11.53 |
| *Pyrrhura cruentata* | VU | 10.86 ± 8.21 | 11.01 ± 5.59 | 8.98 ± 12.20 | 2.56 ± 11.62 |
| *Pyrrhura egregia* | LC | 0.00 ± 0.00 | 0.00 ± 0.00 | 100.00 ± 141.42 | 66.67 ± 57.74 |
| *Pyrrhura frontalis* | LC | 11.22 ± 15.93 | 13.94 ± 11.46 | 35.40 ± 62.48 | 11.92 ± 28.50 |
| *Pyrrhura griseipectus* | CR | 27.97 ± 22.44 | 18.01 ± 14.81 | 9.42 ± 16.69 | 11.06 ± 15.00 |
| *Pyrrhura griseipectus* | EN | 50.37 ± 75.66 | 72.03 ± 77.05 | 64.43 ± 62.63 | 13.87 ± 16.71 |
| *Pyrrhura leucotis* | VU | NaN ± NA | NaN ± NA | NaN ± NA | 15.00 ± NA |
| *Pyrrhura leucotis* | NT | 7.12 ± 7.75 | 10.81 ± 7.18 | 10.14 ± 9.42 | 8.52 ± 17.58 |
| *Pyrrhura leucotis* | LC | 33.81 ± 82.17 | 6.61 ± 10.63 | 0.00 ± 0.00 | 4.67 ± 27.20 |
| *Pyrrhura melanura* | LC | 0.00 ± 0.00 | 3.70 ± 11.11 | 22.22 ± 66.67 | -8.33 ± 42.49 |
| *Pyrrhura molinae* | LC | 8.49 ± 6.69 | 11.11 ± 6.07 | 26.33 ± 28.15 | 13.69 ± 23.10 |
| *Pyrrhura perlata* | VU | 12.89 ± 13.97 | 11.77 ± 5.51 | 24.57 ± 37.52 | 7.60 ± 22.75 |
| *Pyrrhura perlata* | LC | 10.81 ± 15.46 | 32.45 ± 35.37 | 44.87 ± 50.70 | 8.47 ± 23.74 |
| *Pyrrhura perlata coerulescens* | VU | 150.00 ± 300.00 | 0.00 ± 0.00 | 0.00 ± 0.00 | 23.33 ± 59.63 |
| *Pyrrhura perlata coerulescens* | LC | 250.00 ± 353.55 | 0.00 ± 0.00 | 33.33 ± 47.14 | 100.00 ± 0.00 |
| *Pyrrhura picta* | VU | 35.56 ± 110.50 | 24.50 ± 20.00 | 48.11 ± 110.49 | -17.87 ± 47.51 |
| *Pyrrhura picta* | NT | 24.13 ± 26.28 | 14.99 ± 7.65 | 4.27 ± 7.61 | 3.20 ± 24.08 |
| *Pyrrhura picta* | LC | 0.00 ± NA | 13.33 ± NA | 20.00 ± NA | -3.57 ± 5.05 |
| *Pyrrhura roseifrons* | LC | 2.67 ± 8.21 | 19.86 ± 34.20 | 33.84 ± 57.75 | -0.97 ± 41.35 |
| *Pyrrhura rupicola* | NT | 0.00 ± 0.00 | 3.78 ± 5.24 | 49.90 ± 82.08 | 16.61 ± 27.78 |
| *Pyrrhura rupicola* | LC | 0.62 ± 1.98 | 7.67 ± 14.77 | 4.58 ± 10.84 | -2.84 ± 48.04 |
| *Triclaria malachitacea* | NT | 24.04 ± 22.43 | 17.38 ± 17.11 | 1.19 ± 3.46 | -3.69 ± 26.89 |
| *Triclaria malachitacea* | LC | 0.00 ± 0.00 | 9.55 ± 0.64 | 4.55 ± 6.43 | -4.01 ± 11.36 |
